# Boundary-guided cell alignment drives mouse epiblast maturation

**DOI:** 10.1038/s41567-026-03176-9

**Published:** 2026-04-01

**Authors:** Takafumi Ichikawa, Pamela C. Guruciaga, Shuchang Hu, Steffen Plunder, Mei Makino, Marina Hamaji, Anniek Stokkermans, Shinjiro Yoshida, Takashi Hiiragi, Anna Erzberger

**Affiliations:** 1 Department of Developmental Biology, Graduate School of Medicine, Kyoto University, Kyoto, Japan; 2 Institute for the Advanced Study of Human Biology (WPI-ASHBi), Kyoto University, Kyoto, Japan; 3 Cell Biology and Biophysics Unit, European Molecular Biology Laboratory, Heidelberg, Germany; 4 Developmental Biology Unit, European Molecular Biology Laboratory, Heidelberg, Germany; 5 Hubrecht Institute, Royal Netherlands Academy of Arts and Sciences (KNAW), Utrecht, Netherlands; 6 Present Address: Developmental Biology Unit, European Molecular Biology Laboratory, Heidelberg, Germany

**Keywords:** Biophysics, Biological physics

## Abstract

Symmetry breaking and pattern formation occur throughout embryonic development. In early mouse development, a mass of non-polarized epiblast cells in the blastocyst forms the egg cylinder, while cells become apico-basally polarized and build a radial configuration. However, it remains unclear what drives the formation of this tissue architecture. Here we demonstrate that the orientational patterning of epiblast cells is dictated by heterogeneous tissue boundaries, which then defines central lumen positioning. We show that epiblast cells progressively orient perpendicular to the visceral endoderm boundary—which is enriched with the basement membrane protein laminin and the cell surface receptor active integrin β1—but parallel to the extraembryonic ectoderm interface. These orientation dynamics are consistent with general boundary-induced alignment effects in polar materials, with a topological defect predicting the position at which the proamniotic cavity nucleates. The knockout of laminin γ1 and integrin β1 confirms the essential role of adhesion at the epiblast and visceral endoderm boundary. The established epiblast pattern, in turn, facilitates ERK activation—a key cell signalling pathway—to ensure proper epiblast maturation. Together, these findings present the mechanistic basis and functional significance of epiblast tissue patterning.

## Abstract (web summary)

Tissue patterning is essential to development. Now it is shown that boundary cues can drive the patterning of embryonic tissue.

## Main

During embryonic development and homeostasis, cells and tissues repeatedly break symmetry and form patterns. At the tissue scale, patterns can emerge through biochemical and mechanical interactions between cells or at the supracellular level. Morphogen signalling, for example, instructs the formation of tissue patterns with distinct material properties^1–4^. Although these mechanisms have been studied, the effect of tissue boundaries on tissue patterning remains less understood^5–8^, particularly when properties of these boundaries are heterogeneous. Recent studies have begun to investigate how interactions between cells and the extracellular matrix (ECM) deposited at tissue boundaries contribute to tissue formation^9,10^. However, specific mechanisms by which cell–ECM interactions influence cell arrangement, tissue patterning and their subsequent functional consequences remain elusive.

Orientational order—a spatial pattern in living systems—emerges at various scales, from subcellular structures to whole organisms^11–13^. Directional cellular organization, analogous to nematic ordering in liquid crystals^14^, has been observed in two-dimensional (2D) cell cultures, such as neural progenitors^15^ and epithelial cells^16^. However, our understanding of three-dimensional (3D) cell arrangement in tissues remains limited, primarily due to the technical challenges associated with monitoring cellular dynamics within a 3D tissue undergoing pattern formation. Moreover, investigating the potential impact of cell–ECM interactions at the tissue boundary requires approaches to manipulate ECM deposition without disrupting overall tissue architecture.

**Fig. 1:**
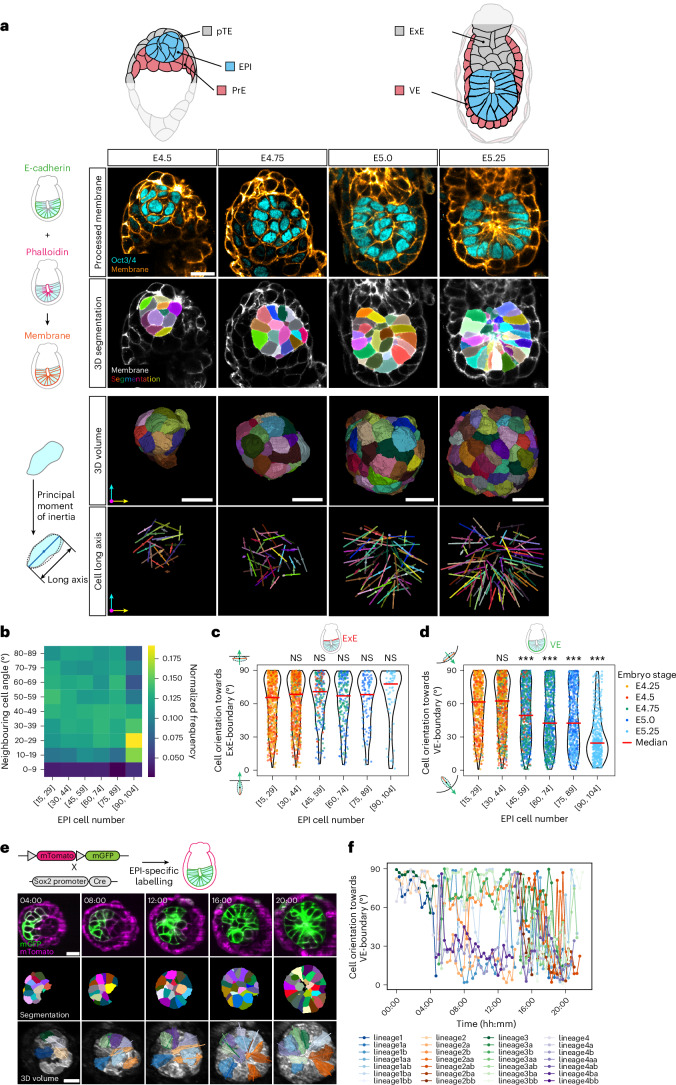
Progressive cell alignment and orientation to the boundary underlie EPI patterning. **a**, Schematic (top) illustrating morphological changes at the cellular and tissue levels during the E4.5–E5.25 window, from blastocyst to cup-shaped egg-cylinder structure with elongated, radially oriented EPI cells (cyan). The EPI is surrounded by pTE/ExE (grey) and PrE/VE (red); other parts of the embryo are shown in light grey. Immunofluorescence images (first row) and the corresponding membrane segmentation (second row) of representative embryos from E4.5 to E5.25, stained for Oct3/4 (EPI; cyan) and cell membrane (orange), generated by combining E-cadherin and phalloidin signals. The third row shows the 3D visualization of segmented cell volumes viewed from a specific angle, with coordinate axes (arrows) indicating the *x*, *y* and *z* dimensions. The bottom row shows the cell long axes extracted by computing principal inertia vectors in three dimensions from the segmented volumes. *n* = 37 (E4.5), 26 (E4.75), 22 (E5.0) and 12 (E5.25) embryos segmented and analysed from at least three independent embryo recovery experiments. **b**, Heat map showing the normalized distribution of angles between the long axes of neighbouring cells, binned in 10° intervals (0°–90°). Data from E4.5 to E5.25 embryos are grouped by the EPI cell number in intervals of 15. Colour intensity represents normalized frequency within each group. Sample sizes by the EPI cell number: [15–29], *n* = 732 cells from 36 embryos; [30–44], 1,226 from 36; [45–59], 890 from 18; [60–74], 1,077 from 17; [75–89], 626 from 8; [90–104], 373 from 4. **c**,**d**, Angle measurement between the cell long axis and the normal vector to the tissue boundary, represented as violin plots with individual data points overlaid. Each plot shows the distribution of angles for EPI cells in contact with the ExE-boundary (**c**) and VE-boundary (**d**). Dot colours indicate the embryo stage. Data are grouped by the EPI cell number, with median values shown by red bars. Sample sizes for **c**: [15–29], *n* = 593 cells from 36 embryos; [30–44], 750 from 35; [45–59], 220 from 15; [60–74], 196 from 12; [75–89], 104 from 8; [90–104], 61 from 5. For **d**: [15–29], *n* = 618 cells from 36 embryos; [30–44], 951 from 35; [45–59], 682 from 15; [60–74], 649 from 12; [75–89], 540 from 8; [90–104], 446 from 5. Mann–Whitney *U*-test (two sided) without correction for multiple comparisons; each group compared with the reference group [15–29 cells]. ***P < 0.001, NS, not significant. Exact *P* values for **d**: [45–59], *P* = 2.35 × 10^−7^; [60–74], *P* = 3.97 × 10^−16^; [75–89], *P* = 3.02 × 10^−13^; [90–104], *P* = 3.54 × 10^−64^. **e**, Time-lapse images of representative Sox2-Cre;mTmG embryos developed in 3D-geec culture system. Green, mG (EPI); magenta, mT (other tissues). Time shown as hours:minutes (hh:mm), with *t* = 00:00 marking the start of imaging. *n* = 5 embryos. **f**, Angle measurement between the cell long axis and the normal vector to the VE-boundary for tracked EPI cells. Each line represents an individual tracked EPI cell, with line colours indicating cell lineages. *n* = 4 initial cells tracked through 2 rounds of cell divisions to 16 cells, from the embryo shown in **e**. Scale bars, 20 µm (Extended Data Fig. 1 and Supplementary Video 1). Source Data

The early mouse embryo provides a model system to investigate the mechanisms of tissue pattern formation^17–19^. Mammalian embryos are derived from the epiblast (EPI) that forms in the blastocyst as an aggregate of non-polarized cells^18,20–23^. Upon implantation and by embryonic day 5.5 (E5.5), the EPI undergoes maturation, a developmental transition during which EPI cells elongate, acquire apico-basal polarity and arrange in a radial manner, while the EPI tissue transforms into the cup-shaped egg-cylinder structure^17,24–26^ (Fig. 1a). During this morphogenesis, the EPI is enveloped by two extraembryonic tissues: the primitive endoderm (PrE) (which differentiates into the visceral endoderm (VE)) and the polar trophectoderm (pTE) (which gives rise to the extraembryonic ectoderm (ExE)). These interactions establish two distinct tissue boundaries with potentially different properties. Indeed, the precise cellular and molecular mechanisms underlying EPI patterning remain elusive, largely due to limited access to the dynamic cellular processes occurring within the uterine tissue.

In this study, building on our recently established method for ex vivo embryo culture and live imaging^24^, together with quantitative image analyses and a theory for boundary-driven polar ordering, we investigate how the EPI tissue pattern is established during mouse peri-implantation development.

## Results

### EPI cells progressively align their long axes with each other and against the tissue boundaries

**Extended Data Fig. 1:**
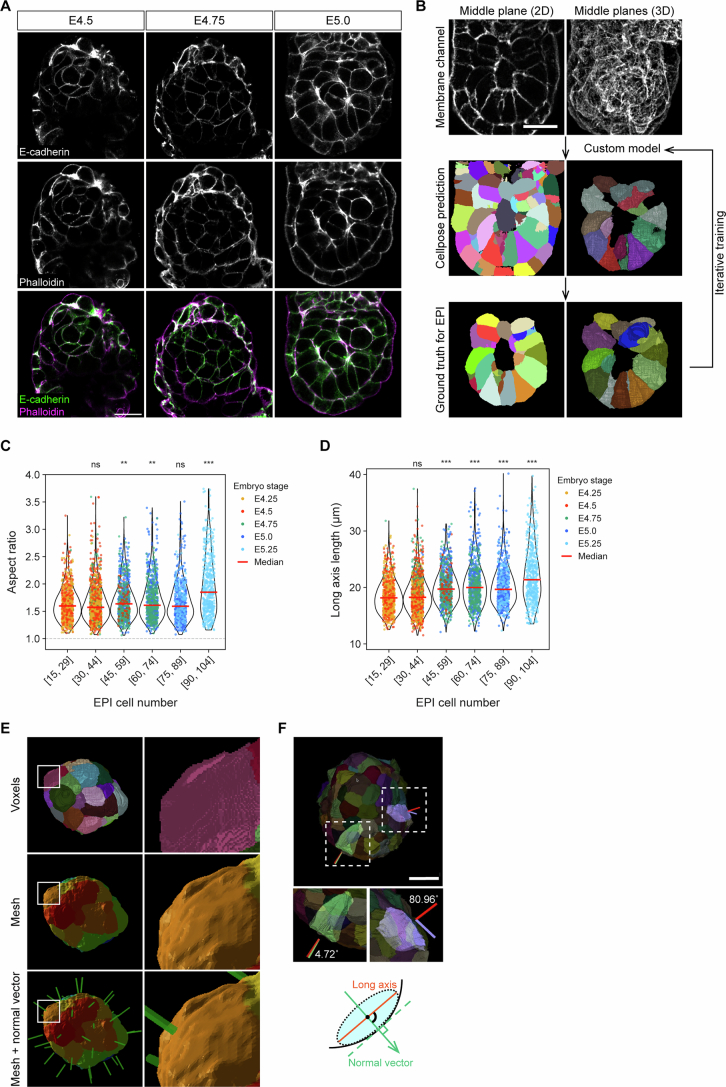
Systematic analysis of EPI cell morphological parameters using 3D membrane segmentation. (**A**) Immunofluorescence images of representative embryos from E4.5 to E5.0, stained for E-cadherin and phalloidin (same samples as Fig. 1a). (**B**) Workflow of 3D membrane segmentation using Cellpose. Membrane channel images (top) were processed with Cellpose (middle). Manual corrections were performed using napari for custom model training (bottom). (**C**) Cell aspect ratio (long axis length / √(middle axis length× short axis length); 1.0 = sphere), shown as violin plots with individual data points. Dot colours indicate embryo stage. Data are grouped by EPI cell number, with median values shown by red bars. Sample sizes: [15–29], *n* = 793 cells from 36 embryos; [30–44], 1259 from 35; [45–59], 783 from 15; [60–74], 775 from 12; [75–89], 644 from 8; [90–104], 495 from 5. Mann-Whitney U test (two-sided) without correction for multiple comparisons; each group compared to the reference group [15–29 cells]. **p < 0.01, ***p < 0.001, ns = not significant. Exact p-values: [45–59], p = 2.81 × 10^−3^; [60–74], p = 3.11 × 10^−3^; [90–104], p = 2.07 × 10^−26^. (**D**) Long axis length, shown as violin plots. Same samples as (**C**). Mann-Whitney U test (two-sided) without correction for multiple comparisons, each group compared to the reference group [15–29 cells]. ***p < 0.001, ns = not significant. Exact p-values: [45–59], p = 6.69 × 10^−25^; [60–74], p = 8.54 × 10^−27^; [75–89], p = 4.33 × 10^−22^; [90–104], p = 1.70 × 10^−44^. (**E**) Surface mesh conversion from voxel-based segmentation (top). Each cell was reconstructed as an individual mesh (middle), and the average normal vector of corresponding mesh vertices was computed (green bars, bottom) (see Methods). (**F**) EPI cell orientation measured as the angle between surface normal and cell long axis (see Methods). Scale bars, 20 µm. See also Fig. 1. Source Data

To understand cellular mechanisms underlying EPI tissue patterning, we first quantitatively characterized EPI cell orientation in mouse embryos developed in utero, at stages from E4.5 to E5.25. To this end, we developed an image analysis pipeline that semiautomatically segments EPI cell membranes in three dimensions based on the combined signal of E-cadherin and phalloidin immunofluorescence (Extended Data Fig. 1a,b). Cell orientation was determined by computing the principal inertia vectors of each segmented 3D cell volume, with the major principal inertia vector defining the cellular long axis (Fig. 1a). This analysis supported our previous observation of EPI cell elongation^24^, with a significant increase in cell aspect ratio and long axis length from early to late stages (Extended Data Fig. 1c,d). Using this 3D quantification, we examined the relationship between the long axes of neighbouring EPI cells as well as between EPI cells and the EPI tissue boundaries. Measurement of the angle between the long axes of neighbouring cells showed that the angle decreases as the embryo develops with an increasing number of EPI cells. This observation indicates that elongated EPI cells progressively align their long axes with each other during egg-cylinder formation (Fig. 1b), suggesting that cell–cell interactions may drive this alignment^24^. In addition, EPI cells establish specific orientations relative to the two distinct tissue–tissue boundaries, namely, with the pTE or ExE (hereafter referred to as the ‘ExE-boundary’), and with the PrE or VE (‘VE-boundary’). We measured the angle between the cellular long axis and the normal vector to each tissue boundary (Extended Data Fig. 1e,f), while the EPI cell number increased from 15 to 104 between E4.25 and E5.25. The median angle at the ExE-boundary increases from 65.57° to 77.80° (Fig. 1c), whereas that at the VE-boundary decreases from 61.53° to 24.50° (Fig. 1d). These data show that EPI cells progressively align themselves parallel to the ExE-boundary and perpendicular to the VE-boundary.

These findings were further corroborated by live imaging of Sox2-Cre;mTmG embryos developing ex vivo using the 3D-gel embedded embryo culture (3D-geec; Fig. 1e)^24^. The analysis of cell orientation dynamics demonstrated that EPI cells progressively establish their orientation perpendicular to the VE-boundary (Fig. 1f). Taken together, our static and dynamic analyses consistently demonstrate that the progressive alignment of elongated EPI cells with each other and relative to the EPI tissue boundaries establishes the EPI tissue-scale alignment pattern.

### Emergence of tissue-scale orientational order in the EPI near the tissue boundary

**Fig. 2:**
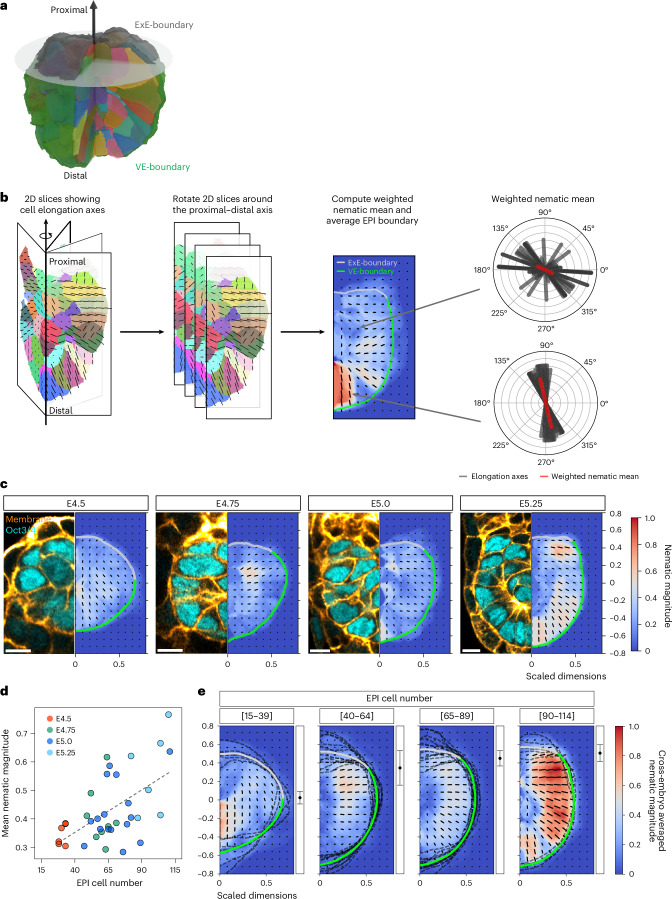
Emergence of tissue-scale cell alignment in the EPI near the tissue boundary. **a**, 3D visualization showing a representative cup-shaped egg-cylinder structure with segmented EPI cells (individual colours), surrounded by the VE-boundary (green) and ExE-boundary (grey). The black arrow indicates the distal–proximal axis. A transverse plane (semitransparent) illustrates a plane that goes through the VE–ExE interface. The approximate rotational symmetry around the distal–proximal axis allows rotated 2D cross-sections through this axis to effectively capture 3D cell alignment patterns. **b**, Schematic illustrating the method used to compute tissue-scale cell alignment patterns. An arrow represents the distal–proximal axis of the EPI tissue, used as the axis of rotation. Individual colours represent 3D segmented cells (Fig. 1), with lines indicating orientations along the long axis and lengths reflecting the anisotropy of the cell (left). Thirty-six sections are reconstructed at 10° rotation intervals around the axis (second from left). With an average EPI boundary shape, the nematic order parameter field was calculated by averaging cell orientations weighted by their elongation across rotational planes (right). Finally, the nematic director field and the magnitude of the nematic alignment were obtained (second from right). **c**, Representative embryo images from E4.5 to E5.25 and their corresponding cell alignment maps. The colour indicates the magnitude of nematic cell alignment, and the lines represent the nematic director vector. Light grey and green thick lines highlight the average ExE-boundary and VE-boundary, respectively. *n* = 6 (E4.5), 8 (E4.75), 16 (E5.0) and 6 (E5.25) embryos analysed. **d**, Scatter plot of the mean nematic magnitude within the EPI region versus the EPI cell number in embryos analysed in **c**. Each dot represents an individual embryo, colour coded by the embryo stage. The dashed line shows linear regression (slope = 0.0030, *R*^2^ = 0.324, *P* = 0.0003). **e**, Cross-embryo average of nematic cell alignment maps grouped by the EPI cell number in intervals of 25. The colour indicates the magnitude of nematic cell alignment, and the lines represent the mean orientation. Light grey and green thick lines highlight the average ExE-boundary and VE-boundary, respectively, and dark grey broken lines indicate the tissue boundary of each embryo. Black dots with error bars at the right indicate the mean ± s.d. position of the interface between ExE-boundary and VE-boundary along the distal–proximal axis. *n* = 6 embryos for EPI cell number [15–39], 12 for [40–64], 13 for [65–89] and 5 for [90–114], used for averaging in each group. Scale bars, 10 µm. 3D rendering in **a** created with Blender 4.5. Source Data

To quantify the tissue-scale orientational order, we developed a computational tool to systematically measure cell alignment patterns across the entire EPI tissue (Fig. 2a). Because the EPI tissue exhibits approximate rotational symmetry around the distal-to-proximal axis, we exploited this symmetry by averaging over rotations about the axis. Specifically, we fixed a 2D cross-section through the distal-to-proximal axis and generated 36 sections at 10° intervals by rotating the EPI section around this axis. Within each section, cell elongation was characterized by the 3D direction of the major principal axis and an anisotropy coefficient ranging from zero (for spheres) to nearly one (for highly elongated cells; Fig. 2b and Methods). We then computed the weighted nematic order parameter field by averaging cell orientations weighted by their elongation across all rotational planes (Fig. 2b and Methods).

This analysis revealed the emergence of a tissue-scale orientational order within the EPI during development (Fig. 2c). In particular, the tissue-scale pattern shows an average perpendicular orientation to the VE-boundary and parallel to the ExE-boundary after E4.75, consistent with our measurements of individual cell orientations (Fig. 1c,d). We quantitatively found a gradual increase in the average nematic magnitude, which measures the degree of cell alignment across the entire EPI region within each embryo (Fig. 2d), in agreement with our initial observations (Fig. 1). To investigate the progression of alignment patterns while accounting for embryo-to-embryo variability, we averaged the nematic field of EPI tissues grouped by their cell number in intervals of 25 (Fig. 2e). Notably, this analysis revealed that the magnitude of nematic alignment is consistently higher near the tissue boundaries compared with the tissue centre, despite the variability between embryos. This robust spatial heterogeneity in alignment strength suggests that tissue–tissue boundaries provide critical instructive cues that not only guide local cell orientation but also influence the global organization of the EPI tissue. These findings indicate a boundary-mediated mechanism for establishing EPI cell organization.

### Surface-induced ordering determines the orientational pattern of the EPI tissue

The progressive alignment of the cells within the tissue and against the tissue boundaries is reminiscent of how anisotropic particles develop orientational order through surface interactions^27–30^. While EPI cells establish their radial orientation, cellular polarity arises from the distinct molecular composition and biophysical organization of the apical and basal domains: the apical domain forms interactions with neighbouring cells (for example, via tight junctions), while the basal domain interacts with the ECM^17,24,26^. These similarities between polar cell alignment in developing tissues and surface-induced ordering of polar materials prompted us to investigate whether the theoretical framework of surface-induced ordering could provide insights into the mechanism of EPI tissue patterning.

**Fig. 3:**
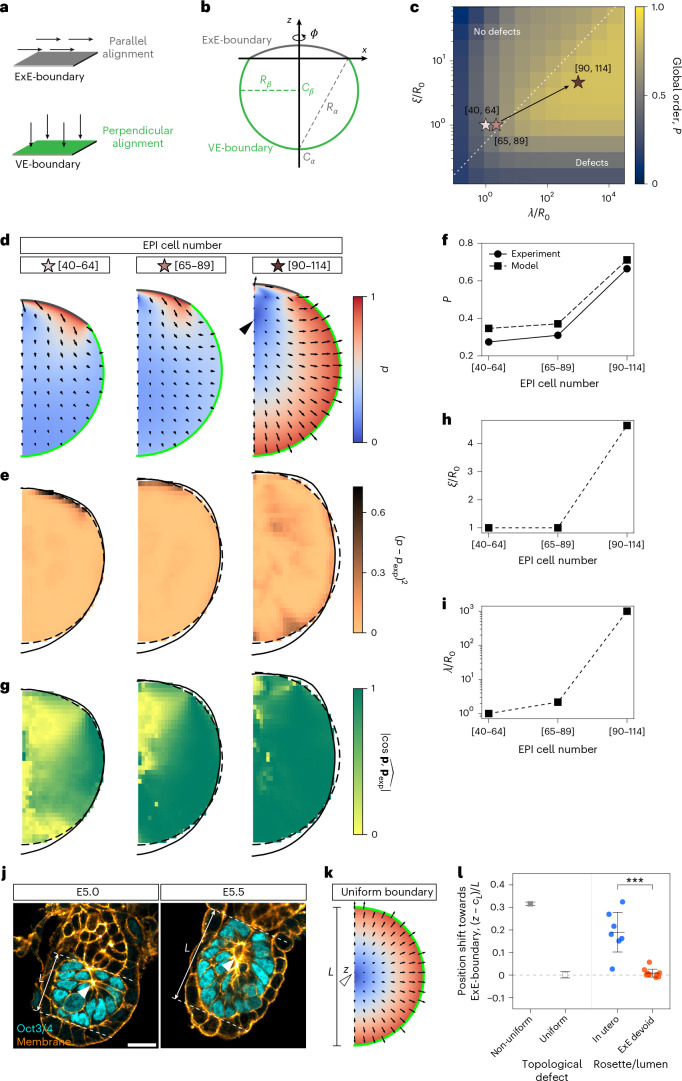
Progressive EPI ordering is driven by boundary-induced alignment. **a**, Schematic illustrating two different surfaces that favour distinct cell alignments: tangential alignment at the ExE-boundary (grey) and perpendicular alignment at the VE-boundary (green). **b**, Schematic of the in silico geometry based on the EPI tissue. Axially symmetric EPI tissue shape was represented by two spherical caps *S*_*μ*_ $(\mu =\alpha ,\beta)$ with radius *R*_*μ*_ and centre *C*_*μ*_. These geometrical parameters were obtained by fitting the ExE-boundary (dark grey) and VE-boundary (dark green) data from Fig. 2e. **c**, Degree of global order *P* of the order parameter field minimizing the effective free energy as a function of the correlation and anchoring lengths (*ξ* and *λ*) relative to the characteristic system size ${R}_{0}=(3{V}_{0}/{4\uppi )}^{1/3}$, where *V*_0_ is the EPI tissue volume. Stars show the position in the parameter space of the three developmental stages studied, obtained by parameter fitting (**d**–**f**). The dotted line marks the transition between defect-free and defect-containing regimes. **d**, Order parameter fields **p** for the best set of parameters for each embryo stage based on EPI cell numbers. The colour map shows their magnitude $p=\left|{\bf{p}}\right|$. The black arrowhead marks the position of the topological defect. **e**, Square difference between the magnitude of the experimentally determined field, *p*_exp_, and that of the fitted order parameter field, *p*, at each point. Data are from the embryos analysed in Fig. 2e. **f**, Degree of global order *P* grows with the stage of the embryos grouped by the EPI cell numbers both in the model (square) and in the experiments (circle). **g**, Absolute degree of alignment between the direction of the experimentally determined field, **p**_exp_, and that of the fitted order parameter field, **p**, at each point (Methods). Note that the directional information was not used during the fitting procedure. **h**,**i**, Material parameters obtained by fitting the theoretical field to the experimentally determined cell orientation field, plotted against the stage of embryos grouped by the EPI cell numbers. Both the correlation length *ξ* (**h**) and the anchoring length *λ* (**i**) relative to the characteristic system size *R*_0_ increase with EPI cell number. **j**, Immunofluorescence images of representative embryos, stained for Oct3/4 (EPI; cyan) and cell membrane (orange). The white arrowheads indicate the rosette structures or a nascent lumen. Tissue length *L* is measured as the distance between the EPI–ExE interface and the distal tip. *n* = 7 embryos. **k**, Order parameter field calculated with the geometry and material parameters of the embryo stage at which the EPI cell number ranges from 90 to 114, with a uniform boundary that has a perpendicular alignment preference. A white arrowhead marks the position of the topological defect at position *z* along the system height *L*. **l**, Scatter plots showing topological defect (model; square) and rosette or lumen (in utero and ExE-devoid embryos; circle) position along the distal–proximal axis, relative to the tissue centre (${c}_{L}=L/2$) and scaled by tissue length *L*. ExE-devoid embryo data adapted from ref. ^31^. *n* = 7 (in utero) and 11 (ExE-devoid) embryos. Black bars indicate mean ± s.d. Student’s *t*-test (two sided), ***P = 1.11 × 10^−5^. Scale bars, 20 µm (Extended Data Figs. 2 and 3). Source Data

Using the Landau–de Gennes approach, which describes orientational order in anisotropic materials^14^, we conceptualized EPI cells as polar particles in a 3D space, constituting a polar fluid characterized by a vector order parameter field **p**. This local order parameter takes a value according to the strength of aligning interactions within the bulk, which competes with the tendency to align with specific directions promoted by the boundaries^31–33^. In particular, we consider an effective free energy functional that takes into account the tendency of cells to align their polarity with that of their neighbours^34,35^, modulated by the correlation length *ξ*. In addition, it phenomenologically incorporates the interactions between EPI cells and the boundaries via soft surface anchoring^33,36^, which penalizes deviations from a preferred value with a strength given by the anchoring length *λ* (Methods). On the basis of our experimental observations, we implemented perpendicular and parallel anchoring terms for the VE-boundary and ExE-boundary, respectively (Fig. 3a). To examine how alignment arises within a geometry that corresponds to that of the EPI tissue, we constructed axially symmetric shapes formed by two spherical caps representing the ExE-boundary and VE-boundary (Fig. 3b and Methods). By minimizing the free energy functional for different correlation and anchoring values, we obtained the corresponding order parameter fields, revealing a critical transition in the alignment pattern when surface anchoring overcomes the cost of bulk distortions. In this surface-dominated regime, singular points (topological defects) with a total charge of +1 appear in the order parameter field, and the global degree of order increases markedly (Fig. 3c)^31^.

**Extended Data Fig. 2:**
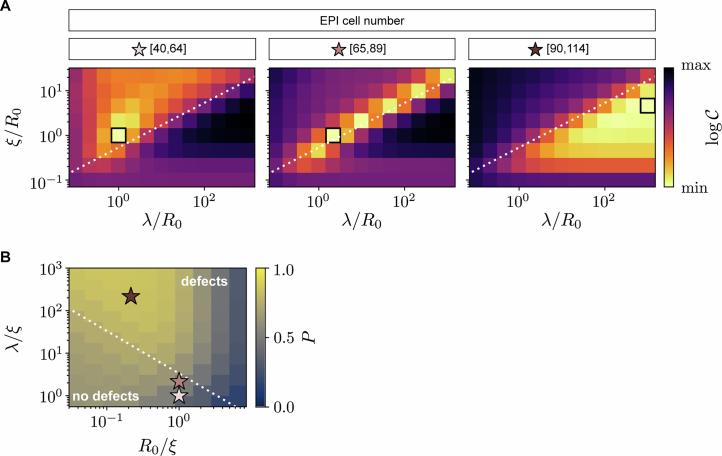
Model parameter optimisation and growth-induced regime transitions. (**A**) Cost function $C$ in terms of the correlation and anchoring lengths, $\xi$ and $\lambda$, relative to the characteristic system size ${R}_{0}$ for all embryo stages based on EPI cell number. Black squares indicate the absolute minimum in each case, corresponding to the set of parameters that best represent the experimental data. The dotted line marks the transition between defect-free and defect-containing regimes. (**B**) Degree of global order $P$ as a function of the ratio of the material length scales, $\lambda /\xi$, and the ratio of the characteristic system size to the correlation length, ${R}_{0}/\xi$. Growth can drive the system from the defect-free into the defect-containing regime if the system size increases faster than the correlation length. Stars show the position in this parameter space of the three developmental stages studied, obtained by fitting in (A). See also Fig. 3.

**Extended Data Fig. 3:**
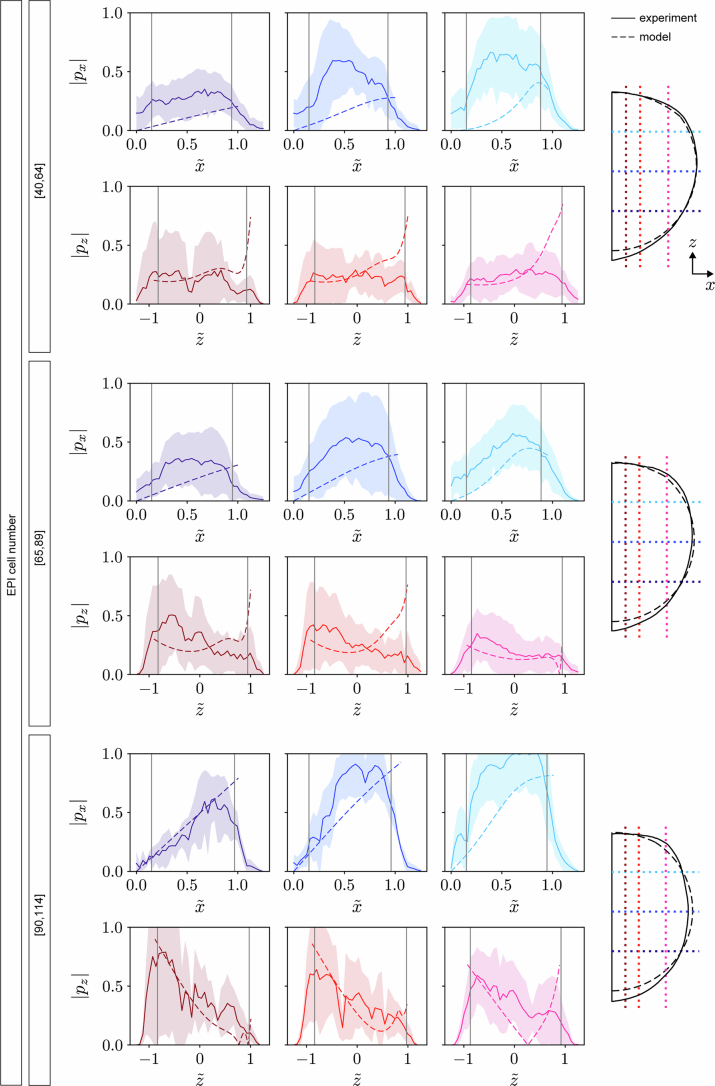
Quantitative comparison of experimental and theoretical orientation fields along tissue cross-sections. Absolute projections $|{p}_{x}|$ and $|{p}_{z}|$ of the experimental and theoretical orientation fields computed along various paths for each embryo stage. Continuous lines and shaded regions represent the mean and standard deviation across embryos. Dashed lines show the values of the fitted order parameter field. Dotted lines with the corresponding colours in the rightmost diagrams indicate the path used for each set of projections. Vertical grey lines delimit the region where the fitting was performed. The spatial coordinates are rescaled such that $\widetilde{x}=0$ represents the symmetry axis and $\widetilde{x}=1$ and $\widetilde{z}=-\mathrm{1,1}$ correspond to the points where each path crosses the experimental average boundary (see Methods). *n* = 12 embryos for EPI cell number [40–64], 13 for [65–89], and 5 for [90–114] (same samples as Fig. 2c–e and Fig. 3d). See also Fig. 3.

To quantify the relative strengths of bulk and surface interactions in the EPI tissue, we withheld the measured average orientations for a subsequent parameter-free comparison, and fitted the correlation and anchoring length parameters using the measured cell elongation pattern (Fig. 2e) as an estimate of the order parameter magnitude at each position. Although the field extracted from the experimental data is nematic, we note that the underlying symmetry of the cells is inherently polar. Assuming that local inversions of apico-basal orientation occur negligibly rarely in the tissue, the nematic order parameter magnitude provides a read-out of the underlying polar order. Hence, we defined the experimental polarity magnitude *p*_exp_ as the strength of the nematic alignment normalized by its maximum across developmental stages and identified the parameter values that minimized the difference between *p*_exp_ and the theoretical magnitude $p=\left|{\bf{p}}\right|$ for each stage (Fig. 3d,e, Extended Data Fig. 2a and Methods). The theoretical results were overall in good agreement with the experimental measurements, with a close correspondence in the global degree of order across different embryonic stages (Fig. 3f). In particular, by the time the EPI cell number reached 90–114 cells, the local direction of the fitted order parameter field was close to that of the corresponding experimental orientation field (Figs. 2e and 3d,g and Extended Data Fig. 3), despite having been fitted using only the local magnitude, providing an independent validation of the model’s suitability. Note that particularly at the earlier stages, the best-fit model shows much higher order near the ExE-boundary than that observed in the experiment, probably due to the simplifying assumption of equal anchoring strength at the two different boundaries. The overall good agreement between the confined polar fluid model and the experimental measurements suggests that orientational cell–cell and cell–boundary interactions are the predominant factors determining the spatial configuration of cells in the EPI, consistent with a fluid-like rheological state of the EPI tissue on the timescale of orientational patterning^24^.

### Maturation of boundary anchoring triggers lumen nucleation

We found that both correlation and anchoring length estimates increased over the course of embryo development, with the increase in the latter much stronger than in the former (Fig. 3h,i). These results suggest that although both cell–cell interactions and cell–boundary interactions strengthen over time, the boundary interactions become dominant at the later stage. Correspondingly, the degree of global order increased from early to late stages in both theory and experiments (Fig. 3f). Moreover, our parameter estimates indicate that the EPI crosses the transition into the surface-dominated, defect-containing regime (Fig. 3c). More generally, such transitions can also be promoted by tissue growth provided that the EPI tissue size increases faster than the correlation length (Extended Data Fig. 2b), although in the EPI, the strengthening of boundary interactions is the main driver. Given that topological defect positions mark the locations of apical-domain clustering and lumen nucleation^31^, these results suggest that rosettes and lumen initiation sites within the EPI should appear once cell–boundary interactions become sufficiently strong to overcome cell–cell alignment effects, that is, the maturation of cell–boundary interactions triggers lumen initiation. More specifically, we predict that molecular determinants of boundary anchoring that mediate cell–ECM interactions should correspondingly increase over the course of development, achieving lumen nucleation at the appropriate developmental stage. Supporting this notion, we consistently observed the nucleation of the proamniotic cavity once the EPI cell number reached approximately 100 cells (Fig. 3j)^24^.

### Boundary heterogeneity guides lumen positioning

Using these experimental estimations based on material parameters, we next investigated how boundary heterogeneity affects internal organization. In particular, we compared order parameter fields with and without an ExE-like interface, the latter having uniform perpendicular anchoring at both boundaries while maintaining other parameters the same. Our simulation reveals that the position of topological defects consistently shifted towards the ExE-boundary in the presence of non-uniform anchoring (Fig. 3d,k), predicting a similar shift in rosette formation and subsequent lumen initiation sites in the EPI tissue. Our experimental observations confirmed this prediction, showing that the rosette and lumen positions were indeed shifted towards the ExE-boundary (Fig. 3l), as opposed to the near-centre lumina observed in the EPI tissue entirely surrounded by the VE, where the ExE had been removed by immunosurgery^31^. Hence, these findings demonstrate that spatial heterogeneity in boundary properties plays a crucial role in guiding tissue architecture in developing embryos.

### Integrin–ECM adhesion is specifically established at the VE-boundary

**Extended Data Fig. 4:**
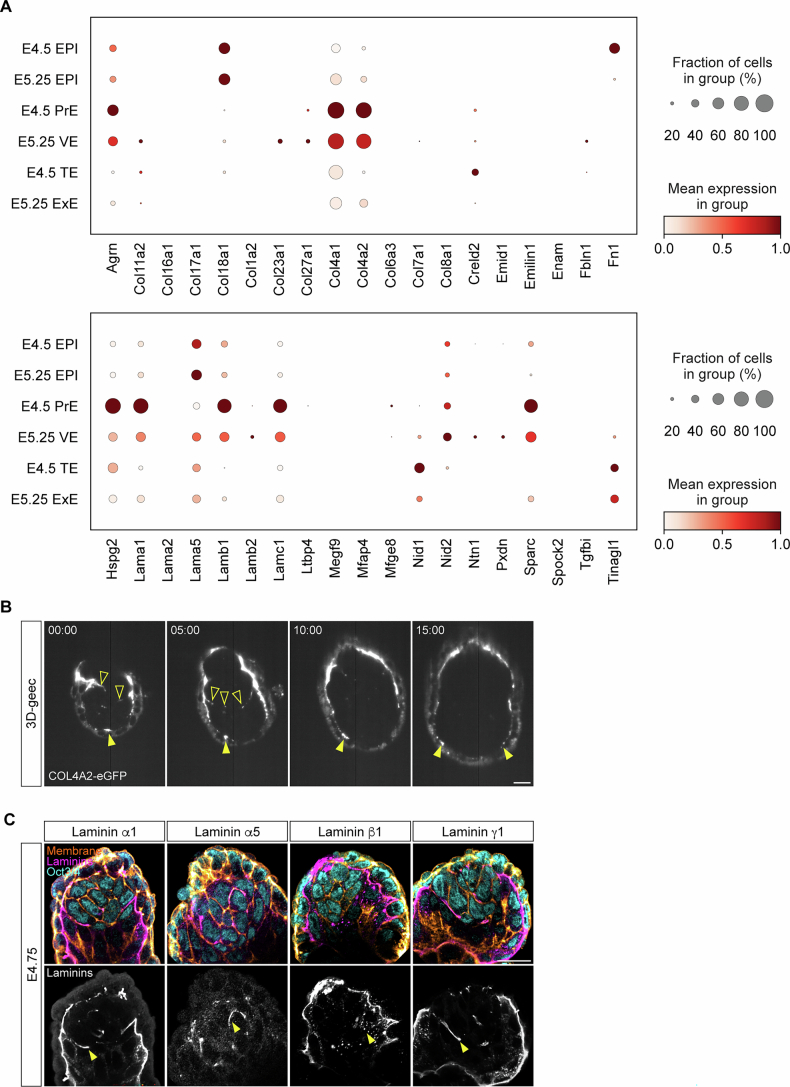
Characterisation of ECM molecules at the tissue boundary. (**A**) Single-cell transcriptome data analysis using a published dataset (^38^Bondarenko et al. 2023). Gene Ontology terms for ECM molecules were used to identify expression levels in single cells isolated from E4.5 and E5.25 embryos. EPI, PrE, VE, TE, and ExE were annotated by Leiden clustering and known markers. (**B**) Time-lapse images of representative Col4a2-eGFP embryos developed in 3D-geec culture system. Time is shown as hours:minutes (hh:mm), with t = 00:00 marking the start of imaging. Signals accumulated at the VE-boundary (yellow arrowhead) and diminished at the ExE-boundary (open arrowhead). *n* = 3 embryos. (**C**) Immunofluorescence images of representative E4.75 embryos stained for laminin α1, laminin α5, laminin β1, and laminin γ1 (magenta), together with cell membrane (orange, derived from E-cadherin and phalloidin), and Oct3/4 (EPI, cyan). *n* = 3 embryos for each laminin subunit from 2 independent litters. Scale bars, 20 µm. See also Fig. 4.

Having established that boundary properties and their heterogeneity influence EPI organization, we next sought to identify the molecular mechanisms that create distinct boundary characteristics. During the course of development, EPI cells progressively elongate (Extended Data Fig. 1c,d) and establish apico-basal polarity, with the basal domain adhering to the ECM via integrins and the apical domain facing towards the future lumen^17,24,26^. The perpendicular alignment of the elongated EPI cells at the VE-boundary therefore positions the basal domain at the boundary interface, where integrin-based adhesion with ECM components such as collagen IV occurs^17,24,37^. To comprehensively characterize the molecular difference between the ExE-boundary and VE-boundary, we first analysed our single-cell transcriptomic data^38^. Genes expressed at least twofold higher in PrE or VE cells compared with TE or ExE cells included *Lama1*, *Lamb1* and *Lamc1*, as well as *Col4a1* and *Col4a2* (Extended Data Fig. 4a).

**Fig. 4:**
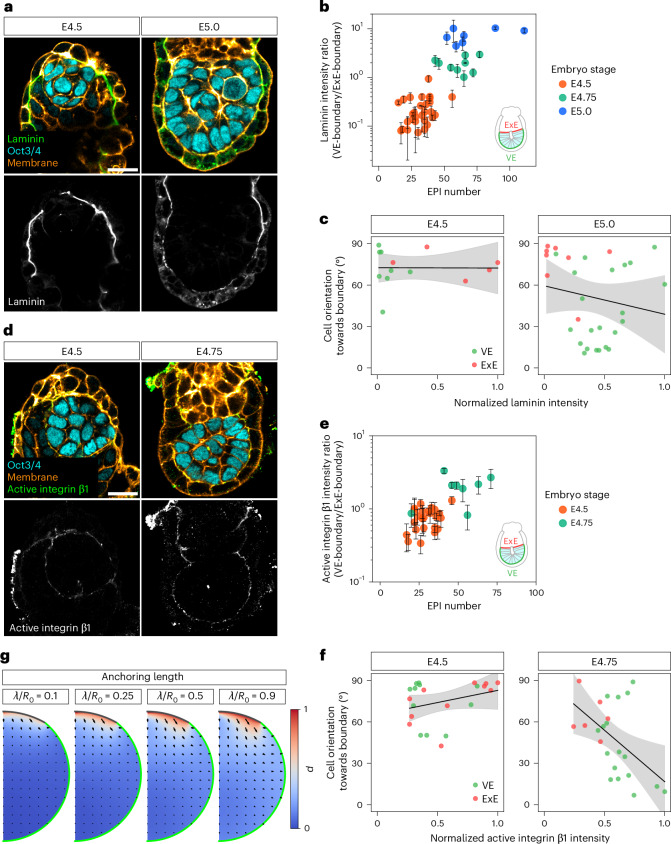
Integrin–laminin adhesion is specifically established at the VE-boundary, correlating with the EPI cell orientation. **a**, Immunofluorescence images of representative embryos from E4.5 to E5.0, stained for laminin (green), Oct3/4 (EPI; cyan) and cell membrane (orange). *n* = 29 (E4.5), 9 (E4.75) and 7 (E5.0) embryos analysed from at least three independent embryo recovery experiments. **b**, Quantification of laminin distribution at the tissue boundary, shown as the ratio of intensity at the VE-boundary to that at the ExE-boundary, based on the embryos in **a**. Each dot represents an individual embryo, plotted against the EPI cell number. Data are presented as mean ± s.d. from three *z* sections (centre ± 6.4 µm) to account for intensity variation (technical replicates). *n* = 29 (E4.5), 9 (E4.75) and 7 (E5.0) embryos. **c**, Scatter plot of EPI cell orientation with respect to surface normal versus normalized laminin intensity at the local tissue boundary, based on the images in **a**. Cells on the middle plane of the 3D EPI volume were used for the analysis. Each dot represents an individual EPI cell in contact with the VE-boundary (green) or ExE-boundary (red). The black lines show linear regression with a 95% confidence interval (shaded band). E4.5: slope = –0.25, *R*^2^ = 4.88 × 10^−5^, *P* = 0.982; E5.0: slope = –20.6, *R*^2^ = 0.033, *P* = 0.336. **d**, Immunofluorescence images of representative embryos from E4.5 to E4.75, stained for active integrin β1 (green), Oct3/4 (EPI; cyan) and cell membrane. *n* = 24 (E4.5) and 8 (E4.75) embryos analysed from at least three independent embryo recovery experiments. **e**, Quantification of active integrin β1 distribution at the tissue boundary, shown as the ratio of intensity at the VE-boundary to that at the ExE-boundary, in embryos shown in **d**. Each dot represents an individual embryo, plotted against the EPI cell number. Data are presented as mean ± s.d. from three *z* sections (centre ± 6.4 µm) to account for intensity variation (technical replicates). *n* = 24 (E4.5) and 8 (E4.75) embryos. **f**, Scatter plot of EPI cell orientation versus normalized active integrin β1 intensity at the local tissue boundary, based on the images in **d**. Cells on the middle plane of the 3D EPI volume were used for the analysis. Each dot represents an individual EPI cell in contact with the VE-boundary (green) or ExE-boundary (red). The black lines show linear regression with a 95% confidence interval (shaded band). E4.5: slope = 17.7, *R*^2^ = 0.092, *P* = 0.171; E4.75: slope = –74.6, *R*^2^ = 0.260, *P* = 0.013. **g**, Order parameter fields with weak anchoring to the surface (λ/*R*_0_ < 1.0) for the average boundary geometry at the EPI cell number stage, [65–89 cells]. Scale bars, 20 µm (Extended Data Fig. 4). Source Data

Next, at the protein level, Col4a2-eGFP^39^ embryos developing in 3D-geec showed GFP signal accumulation at the VE-boundary, whereas it was lost at the ExE-boundary (Extended Data Fig. 4b). Immunostaining of pan-laminin also showed its progressive enrichment at the VE-boundary and diminishing signal at the ExE-boundary (Fig. 4a,b). Laminin-chain-specific antibodies further confirmed that laminin α1, laminin β1 and laminin γ1 accumulated at the VE-boundary, whereas laminin α5 was abundant within the EPI tissue (Extended Data Fig. 4c), in line with our transcriptome analysis (Extended Data Fig. 4a). Moreover, the intensity of the local laminin signal was inversely correlated with the angle between the long axis of the EPI cell and VE-boundary normal (Fig. 4c), supporting a key role for ECM in guiding EPI cell orientation. Consistently, an active form of a major laminin receptor subunit integrin β1 became enriched at the VE-boundary (Fig. 4d,e), again in correlation with EPI cell orientation (Fig. 4f). Collectively, these data demonstrate that integrin–ECM adhesion is specifically established at the VE-boundary, where EPI cells adopt their perpendicular orientation. This suggests that integrin–ECM interactions serve to anchor cells at the boundary and guide cellular alignment, thereby contributing to tissue-scale organization.

### EPI ordering is dependent on laminin γ1 and integrin β1 anchoring

To further investigate the functional role of boundaries in EPI ordering, we first tested in silico the impact of reducing the anchoring strength at the boundary without changing the tissue shape (Fig. 4g). Loss of anchoring resulted in the disruption of polarity alignment, that is, the loss of the orientational order, suggesting the essential role of anchoring to boundaries.

**Fig. 5:**
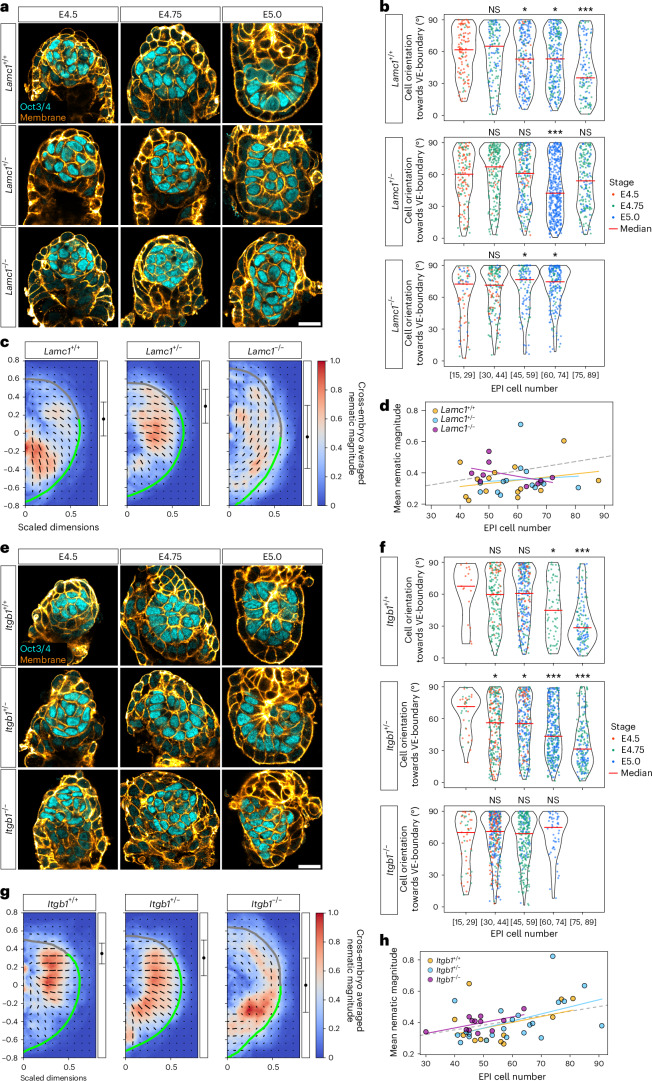
Laminin γ1 and integrin β1 are essential for EPI to build a tissue pattern. **a**, Immunofluorescence images of representative *Lamc1*^+/+^, *Lamc1*^+/−^ and *Lamc1*^−/−^ embryos from E4.5 to E5.0, stained for Oct3/4 (EPI; cyan) and cell membrane (orange). Sample sizes: *Lamc1*^+/+^, *n* = 9 (E4.5), 13 (E4.75), 11 (E5.0); *Lamc1*^+/−^, *n* = 12, 28, 20; *Lamc1*^−/−^, *n* = 4, 11, 9 embryos from 5, 7 and 5 independent *Lamc1*^+/−^ × *Lamc1*^+/−^ litters. **b**, Cell orientation towards the VE-boundary, shown as violin plots with individual data points. Dot colours indicate the embryo stage. Data are grouped by the EPI cell number, with median values shown by red bars. Sample sizes: *Lamc1*^+/+^, *n* = 120 cells from 6 embryos (E4.5), 306 from 8 (E4.75) and 440 from 10 (E5.0); *Lamc1*^+/−^, *n* = 141 from 7 and 503 from 14, 529 from 10; *Lamc1*^−*/*−^, *n* = 66 from 3, 233 from 7 and 250 from 8. Subset of the samples in **a** with sufficient 3D segmentation quality. Mann–Whitney *U*-test (two sided) without correction for multiple comparisons, each group compared with the reference group [15–29 cells]. *P < 0.05, ***P < 0.001. Exact *P* values: *Lamc1*^+/+^ [45–59], *P* = 0.027; [60–74], *P* = 0.026; [75–89], *P* = 4.58 × 10^−7^; *Lamc1*^+/−^ [60–74], *P* = 2.41 × 10^−5^; *Lamc1*^−*/*−^ [45–59], *P* = 0.018; [60–74], *P* = 0.013. **c**, Cross-embryo average of nematic cell alignment maps. Colour indicates nematic alignment magnitude, and lines represent mean orientation. The grey and green thick lines indicate the ExE-boundary and VE-boundary, respectively. Black dots with error bars show the mean ± s.d. position of the interface between ExE-boundary and VE-boundary. *n* = 14 (*Lamc1*^+/+^), 14 (*Lamc1*^+/−^) and 9 (*Lamc1*^−/−^) embryos. **d**, Scatter plot of the mean nematic magnitude versus the EPI cell number analysed in **c**. Each dot represents an individual embryo. The solid lines show linear regression: *Lamc1*^+/+^, slope = 0.0020, *R*^2^ = 0.0690, *P* = 0.364; *Lamc1*^+/−^, slope = 0.0011, *R*^2^ = 0.0078, *P* = 0.764; *Lamc1*^−/−^, slope = –0.0032, *R*^2^ = 0.233, *P* = 0.188. The dashed line shows wild type from Fig. 2d. No significant differences among genotypes (Kruskal–Wallis test, *P* = 0.219). **e**, Immunofluorescence images of representative *Itgb1*^+/+^*, Itgb1* ^+/−^ and *Itgb1*^−/−^ embryos from E4.5 to E5.0, stained for Oct3/4 (EPI; cyan) and cell membrane (orange). Sample sizes: *Itgb1*^+/+^, *n* = 5 (E4.5), 13 (E4.75), 9 (E5.0); *Itgb1*^+/−^, *n* = 13, 28, 26; *Itgb1*^−/−^, *n* = 8, 14, 11 embryos from 5, 8 and 7 independent *Itgb1*^+/−^ × *Itgb1*^+/−^ litters. **f**, Cell orientation towards the VE-boundary, shown as violin plots with individual data points. Dot colours indicate the embryo stage. Data are grouped by the EPI cell number, with median values shown by red bars. Sample sizes: *Itgb1*^+/+^, *n* = 94 cells from 3 embryos (E4.5), 338 from 8 (E4.75), 213 from 4 (E5.0); *Itgb1*^+/−^, *n* = 156 from 5, 477 from 11, 495 from 10; *Itgb1*^−/−^, *n* = 148 from 7, 310 from 11, 254 from 9. Subset of the samples in **e**, with sufficient 3D segmentation quality. Mann–Whitney *U*-test (two sided) without correction for multiple comparisons, each group compared with the reference group [15–29 cells]. *P < 0.05, ***P < 0.001. Exact *P* values: *Itgb1*^+/+^ [60–74], *P* = 0.024; [75–89], *P* = 1.30 × 10^−4^; *Itgb1*^+/−^ [30–44], *P* = 0.013; [45–59], *P* = 0.020; [60–74], *P* = 1.72 × 10^−5^; [75–89], *P* = 1.59 × 10^−9^. **g**, Cross-embryo average of nematic cell alignment maps. Colour indicates the nematic alignment magnitude, and lines represent the mean orientation. The grey and green thick lines indicate the ExE-boundary and VE-boundary, respectively. Black dots with error bars show the mean ± s.d. position of the interface between the ExE-boundary and VE-boundary. *n* = 12 (*Itgb1*^+/+^), 22 (*Itgb1*^+/−^) and 12 (*Itgb1*^−/−^) embryos. **h**, Scatter plot of the mean nematic magnitude versus the EPI cell number analysed in **g**. Each dot represents an individual embryo. The solid lines show linear regression: *Itgb1*^+/+^, slope = 0.0037, *R*^2^ = 0.152, *P* = 0.211; *Itgb1*^+/−^, slope = 0.0045, *R*^2^ = 0.244, *P* = 0.0195; *Itgb1*^−/−^, slope = 0.0035, *R*^2^ = 0.343, *P* = 0.0455. The dashed line shows the wild type from Fig. 2d. No significant differences among genotypes (Kruskal–Wallis test, *P* = 0.324). Scale bars, 20 µm (Extended Data Figs. 5 and 6). Source Data

**Extended Data Fig. 5:**
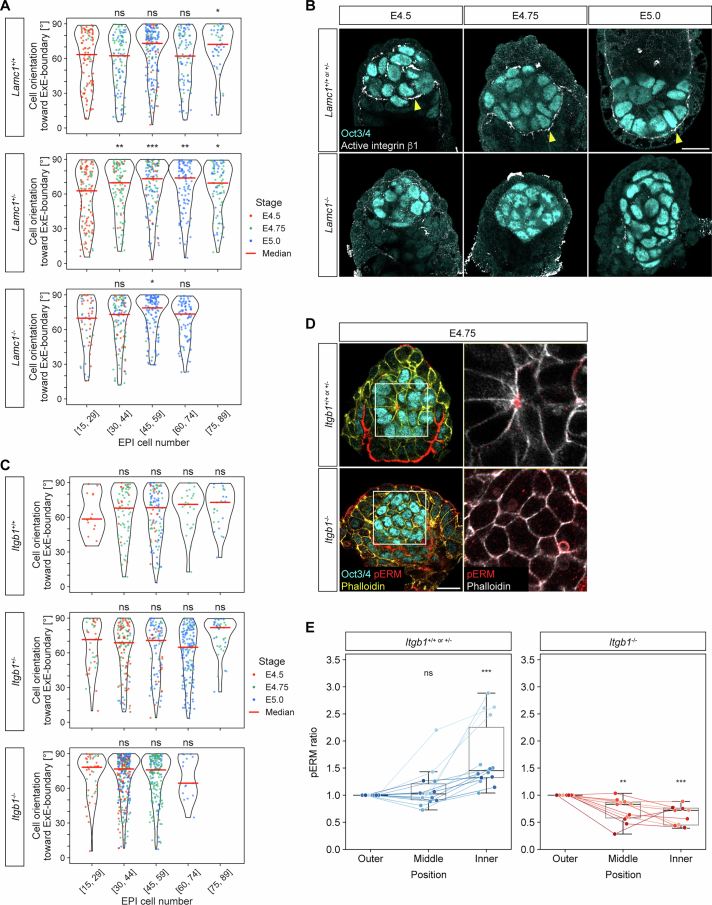
Impacts of *Lamc1* and *Itgb1* genetic perturbations on apico-basal polarisation. (**A**) Cell orientation toward ExE-boundary, shown as violin plots with individual data points. Dot colours indicate embryo stage; red bars show medians. Sample sizes: *Lamc1*^+/+^, *n* = 114 cells from 6 embryos (E4.5), 167 from 8 (E4.75), 247 from 10 (E5.0); *Lamc1*^+/−^, *n* = 132 from 7, 226 from 14, 179 from 10; *Lamc1*^−*/*−^, *n* = 50 from 3, 91 from 7, 230 from 8. Same samples as Fig. 5b. Mann-Whitney U test (two-sided) without correction for multiple comparisons, each group compared to the reference group [15-29 cells]. *p < 0.05, **p < 0.01, ***p < 0.001, ns = not significant. Exact p-values: *Lamc1*^+/+^ [75–89], p = 0.014; *Lamc1*^+/−^ [30–44], p = 0.0066; [45–59], p = 5.36 × 10^−4^; [60–74], p = 0.0019; [75–89], p = 0.011; *Lamc1*^−/−^ [45–59], p = 0.043. (**B**) Immunofluorescence images of representative *Lamc1*^+/+ or +/−^ and *Lamc1*^−/−^ embryos from E4.5 to E5.0, stained for Oct3/4 (EPI, cyan) and active integrin β1 (white). Yellow arrowheads indicate signals at the VE-boundary. Same samples as Fig. 5a. (**C**) Cell orientation toward ExE-boundary, as in (**A**). Sample sizes: *Itgb1*^+/+^, *n* = 46 cells from 3 embryos (E4.5), 132 from 8 (E4.75), 58 from 4 (E5.0); *Itgb1*^+/−^, *n* = 98 from 5, 144 from 11, 212 from 10; *Itgb1*^−/−^, *n* = 129 from 7, 258 from 11, 175 from 9. Same samples as Fig. 5f. Mann-Whitney U test (two-sided) without correction for multiple comparisons, each group compared to the reference group [15-29 cells]. ns = not significant. (**D**) Immunofluorescence images of representative *Itgb1*^+/+ or +/−^ and *Itgb1*^−/−^ embryos at E4.75, stained for Oct3/4 (EPI, cyan), phospho-ERM (red), and phalloidin (yellow). Right panels show enlarged views. Same samples as Fig. 5e. (**E**) Box plots of pERM intensity ratio from three concentric zones, normalised to the outer zone. Individual embryos shown as lines. Box plots show median (centre), 25th–75th percentiles (box), with whiskers extending to 1.5x interquartile range. *n* = 14 (*Itgb*^+/+ or +/−^), 11 (*Itgb*^−/−^) embryos at E4.75. One-sample Wilcoxon signed-rank test (two-sided) comparing each zone to the reference value of 1 (outer). **p < 0.01, ***p < 0.001. ns = not significant. Exact p-values: *Itgb1*^+/+ or +/−^: Inner, p = 1.22 × 10^−4^. *Itgb1*^−/−^: Middle, p = 0.0020; Inner, p = 9.77 × 10^−4^. Scale bars, 20 µm. See also Fig. 5. Source Data

To experimentally test the functional role of boundary anchoring in EPI cell alignment, we genetically perturbed ECM deposition. At the VE-boundary, major laminin chains (laminin α1, β1 and γ1) were present (Extended Data Fig. 4a,c), though *Lama1* and *Lamb1* knockout embryos develop normally until E5.5, presumably due to functional compensation^40,41^. Genetic studies have demonstrated the essential role of laminin γ1 and integrin β1 in the egg-cylinder morphogenetic process^42,43^, in agreement with recent studies using embryo models^17,26^. Here we showed that EPI cells in *Lamc1*^−/−^ embryos were oriented tangentially to the VE-boundary, similar to their alignment at the ExE-boundary, in contrast to *Lamc1*^+/+^ or *Lamc1*^+/−^ controls (Fig. 5a,b and Extended Data Fig. 5a). Moreover, the VE-boundary in *Lamc1*^−/−^ embryos lacked active integrin β1 signal (Extended Data Fig. 5b), indicating that laminin γ1 is required for EPI cells to orient perpendicularly, through integrin β1-mediated anchoring. Tissue-scale analysis further revealed a loss of the polar alignment in *Lamc1*^−/−^ embryos, suggesting a disruption in EPI development (Fig. 5c). In particular, the overall degree of cell alignment, defined as the average nematic magnitude, was comparable between genotypes within the accessible developmental window, indicating that laminin γ1 specifically controls the orientation (Fig. 5d).

Furthermore, EPI cells in *Itgb1*^−/−^ embryos were oriented tangentially to the VE-boundary, unlike the *Itgb1*^+/+^ or *Itgb1*^+/−^ littermates (Fig. 5e,f and Extended Data Fig. 5c), confirming the essential role of integrin β1 in guiding EPI cell orientation at the VE-boundary. Tissue-scale analysis showed the loss of perpendicular alignment with preserved alignment magnitude in *Itgb1*^−/−^ embryos (Fig. 5g,h), resembling theoretical field configurations for full parallel anchoring^31^. Analysis of lumen nucleation by the immunofluorescence of an apical-domain marker, phospho-ERM (pERM), showed that apical-domain convergence was lost in *Itgb1*^−/−^ embryos, with pERM signal enriched at the tissue periphery rather than at the centre (Extended Data Fig. 5d,e), consistent with the theoretical prediction for the lack of topological defects when boundary anchoring is disrupted (Fig. 4g).

**Extended Data Fig. 6:**
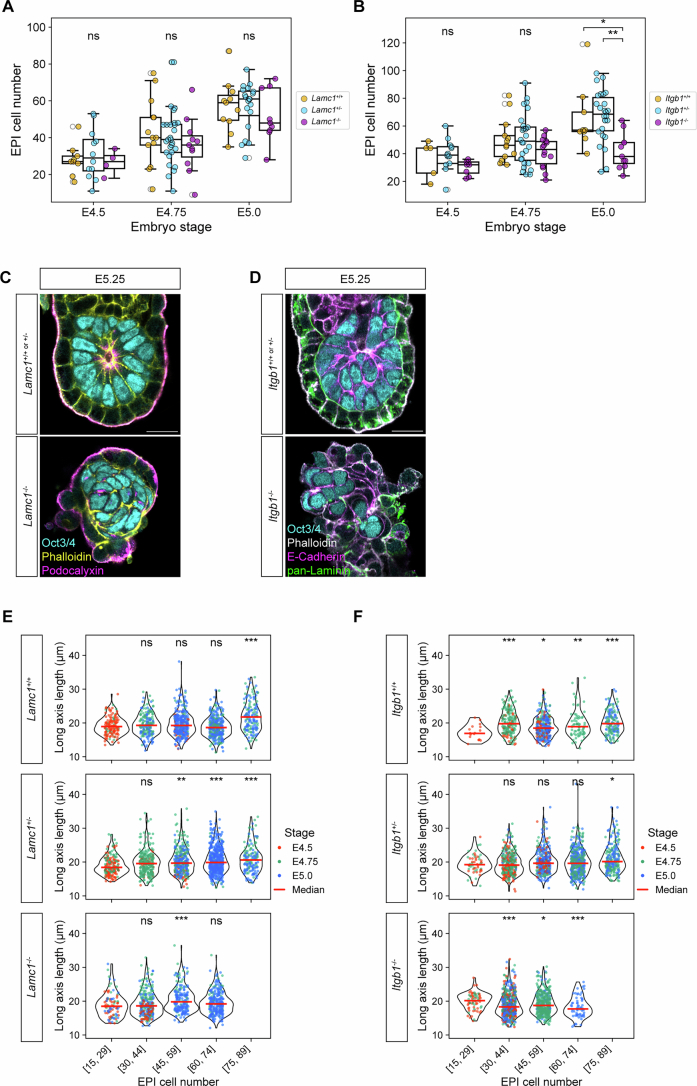
Impacts of *Lamc1* and *Itgb1* genetic perturbations on EPI cell number and morphology. (**A**) Box plots of EPI cell number, showing median (centre), 25th–75th percentiles (box), with whiskers extending to 1.5x interquartile range, with individual embryos shown as dots. Sample sizes: *Lamc1*^+/+^, *n* = 9 (E4.5), 13 (E4.75), 11 (E5.0); *Lamc1*^+/−^, *n* = 12, 28, 20; *Lamc1*^−/−^, *n* = 4, 11, 9 embryos from 5, 7, and 5 independent *Lamc1*^+/−^ × *Lamc1*^+/−^ litters. One-way ANOVA with Tukey’s post hoc test, ns = not significant. (**B**) Box plots of EPI cell number, as in (**A**). Sample sizes: *Itgb1*^+/+^, *n* = 5 (E4.5), 13 (E4.75), 9 (E5.0); *Itgb1*^+/−^, *n* = 13, 28, 26; *Itgb1*^−/−^, *n* = 8, 14, 11 embryos from 5, 8, and 7 independent *Itgb1*^+/−^ × *Itgb1*^+/−^ litters. One-way ANOVA with Tukey’s post hoc test. *p < 0.05, **p < 0.01, ns = not significant. At E5.0, *Itgb1*^+/+^ vs *Itgb1*^−/−^, p = 0.017; *Itgb1*^+/−^ vs *Itgb1*^−/−^, p = 0.001. (**C**) Immunofluorescence images of representative *Lamc1*^+/+ or +/−^ and *Lamc1*^−/−^ embryos at E5.25, stained for Oct3/4 (EPI, cyan), phalloidin (yellow), and podocalyxin (magenta). *n* = 28 (*Lamc1*^+/+ or +/−^), 6 (*Lamc1*^−/−^) embryos from 4 independent *Lamc1*^+/−^ × *Lamc1*^+/−^ litters. (**D**) Immunofluorescence images of representative *Itgb1*^+/+ or +/−^ and *Itgb1*^−/−^ embryos at E5.25, stained for Oct3/4 (EPI, cyan), pan-laminin (green), and phalloidin (yellow). *n* = 13 (*Itgb1*^+/+ or +/−^), 2 (*Itgb1*^−/−^) embryos from 2 independent *Itgb1*^+/−^ × *Itgb1*^+/−^ litters. (**E**) Long axis length measurement, shown as violin plots with individual data points. Dot colours indicate embryo stage; red bars show medians. Sample sizes: *Lamc1*^+/+^, *n* = 163 cells from 6 embryos (E4.5), 397 from 8 (E4.75), 571 from 10 (E5.0); *Lamc1*^+/−^, *n* = 199 from 7, 583 from 14, 637 from 10; *Lamc1*^−*/*−^, *n* = 75 from 3, 277 from 7, 462 from 9. Mann-Whitney U test (two-sided) without correction for multiple comparisons, each group compared to the reference group [15-29 cells]. *p < 0.05, **p < 0.01, ***p < 0.001, ns = not significant. Exact p-values: *Lamc1*^+/+^ [30–44], p = 0.345; [45–59], p = 0.189; [60–74], p = 0.236; [75–89], p = 8.72 × 10^−9^. *Lamc1*^+/−^ [30–44], p = 0.051; [45–59], p = 1.00 × 10^−3^; [60–74], p = 5.02 × 10^−6^; [75–89], p = 7.42 × 10^−6^. *Lamc1*^−/−^ [30–44], p = 0.539; [45–59], p = 4.07 × 10^−4^; [60–74], p = 0.059. (**F**) Long axis length measurement, as in (**E**). Sample sizes: *Itgb1*^+/+^, *n* = 110 cells from 3 embryos (E4.5), 399 from 8 (E4.75), 251 from 4 (E5.0); *Itgb1*^+/−^, *n* = 200 from 5, 560 from 11, 618 from 10; *Itgb1*^−/−^, *n* = 209 from 7, 470 from 11, 360 from 9. Mann-Whitney U test (two-sided) without correction for multiple comparisons, each group compared to the reference group [15-29 cells]. *p < 0.05, **p < 0.01, ***p < 0.001, ns = not significant. Exact p-values: *Itgb1*^+/+^: [30–44], p = 2.36 × 10^−4^; [45–59], p = 0.015; [60–74], p = 8.97 × 10^−3^; [75–89], p = 2.34 × 10^−4^. *Itgb1*^+/−^: [30–44], p = 0.845; [45–59], p = 0.335; [60–74], p = 0.518; [75–89], p = 0.022. *Itgb1*^−/−^: [30–44], p = 5.65 × 10^−4^; [45–59], p = 0.022; [60–74], p = 3.71 × 10^−4^. Scale bars, 20 µm. See also Fig. 5. Source Data

The phenotype in both *Lamc1*^−/−^ and *Itgb1*^−/−^ embryos represents a fundamental developmental defect rather than a developmental delay, supported by a progressive decline in EPI cell numbers, severe disorganization and reduced embryo recovery rates by E5.25 (Extended Data Fig. 6a–d). Together, these results demonstrate that integrin β1–laminin γ1-mediated adhesion is essential for EPI cells to form the polar alignment perpendicular to the VE-boundary, and for EPI tissue ordering and subsequent development, including lumen formation.

### EPI tissue pattern facilitates activation of ERK pathway in EPI cells

**Fig. 6:**
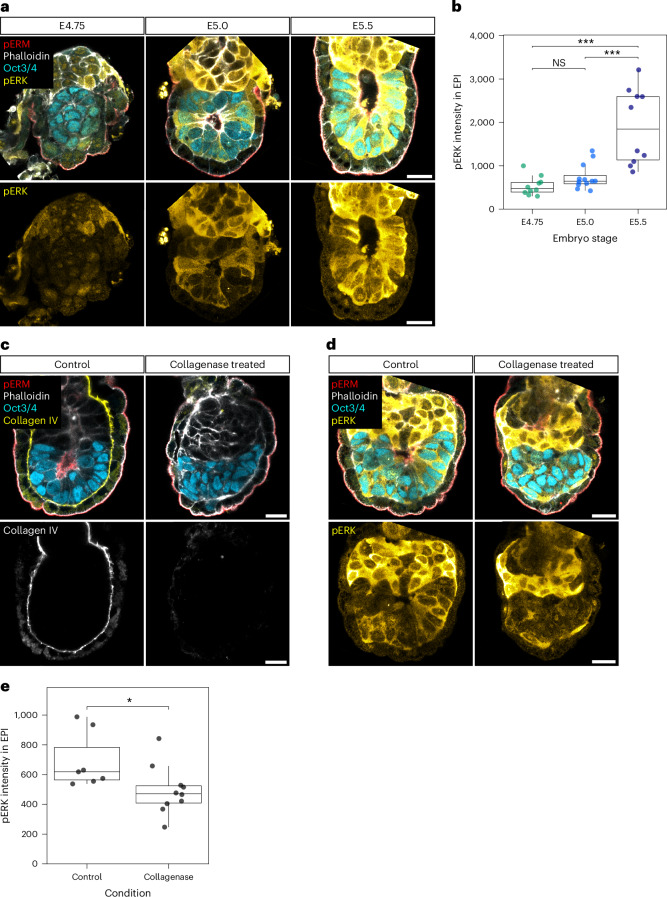
EPI tissue patterning facilitates ERK activation in EPI cells. **a**, Immunofluorescence images of representative embryos from E4.75 to E5.5, stained for pERM (apical, red), phalloidin (white), Oct3/4 (EPI; cyan) and pERK (yellow). *n* = 21 (E4.75), 18 (E5.0) and 13 (E5.5) embryos analysed from 3 independent embryo recovery experiments. **b**, Quantification of mean pERK signal intensity within the EPI tissue. Each dot represents an individual embryo. *n* = 10 (E4.75), 12 (E5.0) and 10 (E5.5) embryos measured from the embryos shown in **a**. Box plots show the median (centre line), 25th–75th percentiles (box bounds) and whiskers extending to 1.5× the interquartile range. Points beyond the whiskers indicate outliers. One-way analysis of variance (*P* = 4.56 × 10^−6^) with Tukey’s post hoc test. ***P < 0.001. Exact *P* values: E4.75 versus E5.0, *P* = 0.64; E4.75 versus E5.5, *P* = 1.00 × 10^−5^; E5.0 versus E5.5, *P* = 6.01 × 10^−5^. **c**, Immunofluorescence images of representative control and collagenase-treated embryos, stained for pERM (apical, red), phalloidin (white), Oct3/4 (EPI; cyan) and collagen IV (yellow). *n* = 8 (control) and 8 (collagenase-treated) embryos from 2 independent embryo culture experiments. **d**, Immunofluorescence images of representative embryos cultured for 16 h in control or collagenase-containing medium, stained for pERM (apical; red), phalloidin (white), Oct3/4 (EPI; cyan) and pERK (yellow). *n* = 14 (control) and 17 (collagenase-treated) embryos cultured from 3 independent experiments. **e**, Quantification of mean pERK signal intensity within the EPI tissue. Each dot represents an individual embryo. *n* = 7 (control) and 10 (collagenase-treated) embryos measured from the embryos shown in **d**. Box plots show the median (centre line), 25th–75th percentiles (box bounds) and whiskers extending to 1.5× the interquartile range. Points beyond the whiskers indicate outliers. Mann–Whitney *U*-test (two sided); *P = 0.014. Source Data

The reduced EPI cell numbers observed in *Lamc1*^−/−^ and *Itgb1*^−/−^ embryos (Extended Data Fig. 6a,b) prompted us to test whether proper EPI cell alignment may be linked to cell differentiation and/or proliferation through the activation of signalling pathways, such as the extracellular signal-regulated kinase (ERK) cascade. Immunofluorescence for phosphorylated ERK1/2 (pERK) in E4.75–5.5 embryos showed a progressive increase in the overall pERK signal within the EPI with notable cell-to-cell heterogeneity, in addition to those in ExE cells (Fig. 6a,b)^44,45^. Moreover, the disruption of EPI cell alignment by 16-h treatment with collagenase that selectively degrades collagen IV at the VE-boundary (Fig. 6c) resulted in a reduction in the overall pERK levels compared with controls (Fig. 6d,e). Together, these findings show that EPI tissue patterning promotes the activation of ERK signalling in EPI cells, highlighting the critical role of cell alignment patterning in regulating signalling pathways crucial for cell differentiation and proliferation.

## Discussion

This study reports that boundary-induced cell alignment drives the emergence of polarized architecture in the mouse EPI tissue. Our experiments, in combination with a theory of polar fluid ordering^31^, consistently show that cell–ECM interactions guide the EPI cell alignment, which, in turn, facilitates the functional maturation of the EPI to allow lumen formation and ERK activation. Using 3D quantifications of cell orientation across developmental stages, we identified how the biophysical parameters associated with cell–boundary and cell–cell interactions of the EPI tissue change over the course of development, driving the transition that enables the nucleation of the proamniotic cavity.

The specific boundary condition defined by the localized expression of laminin at the VE-boundary is essential for EPI cell alignment. This finding is in agreement with earlier studies showing the role of integrin β1 and laminin γ1 in embryonic development through E5.5 (refs. ^42,43,46^). Moreover, we demonstrate that the differential expression of ECM components between the ExE-boundary and VE-boundary determines the characteristic EPI tissue architecture in the egg cylinder, indicating the importance of the embryonic–extraembryonic tissue interface. From a theoretical perspective, it is worth noting that activity in confined systems itself can induce anchoring at boundaries^47–50^, leading to the formation of defects and holes^51–53^. In our system, ECM proteins play a decisive role by converting the otherwise parallel anchoring into perpendicular alignment at the VE-boundary. Our theoretical framework, based on the physics of boundary-induced alignment, successfully captures the complex changes in cellular organization over time, despite its simplicity and reliance on just two key material parameters.

Moreover, our study couples changes in tissue boundary interactions to the formation of lumen initiation sites within the EPI tissue^31^. These sites appear where the predicted orientation field features topological defects^31^—localized singularities that determine the collective properties of ordered systems and guide diverse biological functions, including cell extrusion, protrusion and morphogenesis^15,16,54^. The heterogeneous boundary conditions of the EPI create lumen-inducing 3D topological defects when anchoring strength overcomes the effects of bulk interactions, suggesting that the maturation of the tissue–ECM interface coordinates lumen formation. In particular, we show both in silico and in vivo that the ExE-boundary brings the nucleating lumen position closer to the ExE, thereby potentially contributing to embryo symmetry breaking through lumen-associated key signalling pathways, such as Nodal^25^ and BMP^55^.

Although the role of FGF–ERK signalling has been studied in the EPI–PrE segregation in the blastocyst^20,21,56–58^ and in the ExE in post-implantation development^44,45^, its activity in the EPI in the peri-implantation embryo remained elusive. Our findings for the ERK signalling in the EPI are consistent with studies in embryonic stem cells that suggested the role of FGF–ERK signalling in driving the transition from naïve to formative pluripotency^59–62^. This suggests that the exit from the naïve state is coupled to EPI tissue organization during peri-implantation development^63^. Furthermore, cell-to-cell heterogeneity in ERK activation is in line with our previous observation of Dusp4 (ref. ^24^), which negatively feeds back on FGF signalling^64^. These findings provide a framework for future investigation into the regulatory mechanisms and dynamics of ERK activation and exit from naïve pluripotency in peri-implantation EPI.

During EPI patterning in the peri-implantation mouse embryo, EPI cells undergo dynamic cellular changes, including elongation and apico-basal polarization^17,24–26^, an emergent property of mechano-chemical feedback. These processes are fundamentally coupled with cell alignment and functional maturation, as evidenced by the coordinated phenotypes, including cell elongation, observed in *Lamc1*^−/−^ and *Itgb1*^−/−^ embryos (Fig. 5 and Extended Data Fig. 6e,f). Integrin–ECM interactions probably contribute to this coupling through oriented cytoskeletal organization^65,66^. Due to technical challenges in tracking polarity markers over time in the developing EPI, our current analysis infers the magnitude of polarity alignment from 3D cell shape measurements. Future studies will be valuable to gain insights into the contribution of cell polarization dynamics and its potential role in cell elongation during this tissue patterning process.

## Methods

### Mouse work

All animal work was performed in the Institute of Laboratory Animals, with permission from the Animal Research Committee, Graduate School of Medicine (approval number MedKyo 23065), and the Committee for Safety Control of Recombinant DNA Experiments, Kyoto University (approval number 230029). Institute of Laboratory Animals is operated according to the Regulations on Animal Experimentation at Kyoto University. All mice were maintained in specific-pathogen-free conditions with a 14–10-h light–dark cycle and used for experiments at the age of 8 to 30 weeks.

### Mouse lines and genotyping

The following mouse lines were used in this study: F1 hybrid strain between C57BL/6NCrSlc and C3H/HeSlc (B6C3F1/Slc; Japan SLC) as wild type, mTmG^67^, *Sox2-Cre*^68^, *Itgb1*^*tm1Efu (floxed)*^^69^, *Lamc1*^*tm1Str (floxed)*^^70^ and *Col4a2-eGFP*^39^. To generate *Itgb1*^*+/*−^ and *Lamc1*^*+/*−^ mice, *Itgb1*^*floxed/floxed*^ and *Lamc1*^*floxed/floxed*^ females were crossed with *ZP3-Cre* ^*tg/+*^ males, followed by crossing between *Itgb1*^*floxed/+*^;*Zp3-Cre*^*tg/+*^ and *Lamc1*^*floxed/+*^;*Zp3-Cre*^*tg/+*^ females and B6C3F1 males, respectively^71^. Standard genotyping procedures were used to genotype transgenic mice (Supplementary Table 1 lists the primers and polymerase chain reaction (PCR) product sizes).

### Embryo recovery

To obtain mouse embryos, mice were naturally mated, and the midpoint of the light period on the day when a vaginal plug was detected was defined as embryonic day 0.5 (E0.5). Recovery of embryos was performed under a stereomicroscope (ZEISS, Stemi 508) equipped with a thermo plate (Tokai Hit, TPi-STMX) heated at 37 °C. Peri- and post-implantation embryos were recovered from dissected uteri in a dissection medium (Dulbecco’s modified Eagle’s medium (Gibco, 11880028) supplemented with 15% heat-inactivated fetal bovine serum (PAA, A15-080), 2 mM of GlutaMAX (Gibco, 35050061), 10 mM of HEPES (Sigma, H0887), 25 units ml^−1^ of penicillin and 25 µg ml^−1^ of streptomycin (Gibco, 15070063)). Peri-implantation embryos loosely adherent to the uterine luminal epithelium were isolated by opening along with the mesometrial side of the uterus, followed by a gentle touch using fine forceps (Dumont, No. 5). Sites of embryo adherence can be identified by locally expanding tissue undergoing decidualization. Recovery of post-implantation embryos was performed as described previously^24^. The Reichert’s membrane of the post-implantation embryos was removed using Dentronics No. 32 needles (Handaya, HS-2739B) for subsequent immunofluorescence staining. Recovered embryos were handled using an aspirator tube (Sigma, A5177) equipped with a pulled glass micropipette, 100 μl (Drummond, 2-000-1000) and cultured in an incubator (PHC, MCO-170AICUV) with a humidified atmosphere of 5% CO_2_ at 37 °C.

### 3D-gel embedded embryo culture

3D-geec was performed as described previously^24^. Briefly, mural trophectoderm was microsurgically removed from E4.5 embryos using Dentronics No. 32 needles. Mural-trophectoderm-removed embryos were then embedded in the gel droplet composed of 3.0 mg ml^−1^ of growth-factor-reduced Matrigel (Corning, 356230, lot number 7345012) and 0.3 mg ml^−1^ of collagen I (Corning, 354236, lot number 2055001), diluted in a basal medium (advanced Dulbecco’s modified Eagle’s medium/F-12 (Gibco, 12634010) supplemented with 2 mM of GlutaMAX, 25 units ml^−1^ of penicillin and 25 µg ml^−1^ of streptomycin). After 30 min of incubation in the incubator, the gel was solidified, and 50 µl of prewarmed IVC1 medium^17^ was added to cover the gel. To degrade collagen IV, NP-collagenase (Nippi, 892461) was added to the IVC1 medium at 500 µg ml^−1^.

### Single-embryo genotyping

Transgenic mutant embryos were genotyped retrospectively after imaging. Single embryos were transferred using a mouth pipette from the imaging dish into PCR tubes containing 10 μl of lysis buffer composed of Ex Taq buffer (Takara, RR006A) supplemented with 0.2 mg ml^−1^ of Proteinase K (Sigma, P2308). Embryos in the lysis buffer were incubated at 55 °C for 1 h and then at 96 °C for 10 min. Five microlitres of the genomic DNA lysate was subjected to PCR using Ex Taq.

### Immunofluorescence staining and imaging

Embryos were fixed with 4% paraformaldehyde (Fujifilm Wako, 166-23251) in PBS for 20 min (E4.5–5.0) or 30 min (E5.25–5.5) at room temperature and subsequently permeabilized with 0.5% Triton X-100 (Nacalai, 12967-32) in PBS for 30 min at room temperature with gentle agitation. Embryos were incubated in a blocking buffer (3% BSA (Sigma, A9647) and 0.05% Triton X-100 in PBS) overnight at 4 °C with gentle agitation. Embryos were then incubated with primary antibodies diluted in the blocking buffer overnight at 4 °C or 2 h at room temperature. After washing with the blocking buffer, embryos were further incubated with secondary antibodies diluted in the blocking buffer for 2 h at room temperature. Phalloidin staining was performed simultaneously with the secondary antibody staining, using Alexa Fluor Plus 405 Phalloidin (Invitrogen, A30104) diluted at 1:400. Finally, the stained embryos were transferred into PBS droplets overlaid with mineral oil, on a 35-mm glass-base dish (IWAKI, 3970-035) for imaging.

Primary antibodies against Oct3/4 (Santa Cruz Biotechnology, sc-5279 AF647), E-cadherin (BD Biosciences, 560064), active integrin β1 (9EG7, BD Biosciences, 553715) and phosphorylated ERK1/2 (p44/42 MAPK; Cell Signaling, 4370) were diluted at 1:100. Primary antibodies against laminin (Novus-Biologicals, NB300-144), pERM (Cell Signaling, 3726) and podocalyxin (R&D Systems, MAB1556) were diluted at 1:200. Laminin-chain-specific antibodies were used as described previously^71^. Secondary antibodies, donkey anti-rabbit IgG Alexa Fluor Plus 488 (Invitrogen, A32790), donkey anti-rabbit IgG Alexa Fluor Plus 555 (Invitrogen, A32794) and donkey anti-rat IgG Alexa Fluor Plus 488 (Invitrogen, A48269) were used at 1:200.

Images of immunostained embryos were acquired using an LSM980 microscope equipped with a C-Apochromat ×40/1.2-numerical-aperture water-immersion objective (ZEISS), with Airyscan 2 Multiplex CO-8Y mode. Raw Airyscan images were post-processed by ZEN Blue software (ZEISS). The image voxel size after the Airyscan processing was 0.0823 × 0.0823 × 0.1600 µm^3^ (*x* × *y* × *z*).

### Light-sheet live imaging

3D-geec embryos were live imaged using an inverted light-sheet microscope (Bruker, Luxendo, InVi SPIM), as described previously^24^. Briefly, embryos were embedded in 15 µl of gel mix within a V-shaped sample holder attached to a transparent FEP foil, carefully positioned so that they are in proximity but do not adhere to the foil, which could disrupt morphogenesis via cell spreading. After gel solidification, embryos were immersed in 75 µl of IVC1 medium and further covered with 200 µl of mineral oil to prevent evaporation. The sample holder was mounted in an environmentally controlled incubation box with 5% CO_2_ and 5% O_2_ at 37 °C.

InVi SPIM was equipped with a Nikon ×25/1.1-numerical-aperture water-immersion detection objective and a Nikon ×10/0.3-numerical-aperture water-immersion illumination objective. The illumination plane and focal plane were aligned before the imaging session and maintained during the imaging. Images were taken every 20 min by a complementary metal–oxide–semiconductor camera (Hamamatsu, ORCA-Flash4.0 V2) with the line-scan mode in LuxControl (Luxendo). The imaged volume was 212.99 × 212.99 × 200 µm^3^ with a physical voxel size of 0.104 × 0.104 × 1.000 µm^3^. The lasers and filters used were 488 nm and BP525/50 and 561 nm and LP561 to image GFP and tdTomato fluorophores, respectively. The exposure time for each plane was set to 30 ms.

### Simulations of surface-induced order in a polar fluid

We consider a polar fluid in a space *Ω* with volume *V*_0_ confined by the surface *∂Ω*. This system can be characterized by a local order parameter **p** (**r**), which defines the global degree of order $P={\int }_{{\varOmega }}{\rm{d}}V|{\bf{p}}|/{V}_{0}$ and minimizes the free energy functional

1 $$F[{\bf{p}}]={\int }_{\varOmega }{\rm{d}}V[{f}_{{\rm{R}}}({\bf{p}})+{f}_{{\rm{D}}}({\bf{p}},\,\nabla {\bf{p}})]+{\int }_{\partial \varOmega }{\rm{d}}S{f}_{{\rm{S}}}({\bf{p}};{{\bf{p}}}_{0}).$$

The first term corresponds to the bulk energy, with a restoring term ${f}_{{\rm{R}}}=a{\left|{\bf{p}}\right|}^{2}/2$ with ${\alpha}\gtrsim 0$ (that is, the system is in the isotropic phase) and a distortion energy density ${f}_{{\rm{D}}}={k}_{0}{\left(\nabla {\boldsymbol{\cdot }}{\bf{p}}\right)}^{2}/2+{k}_{2}{\left[\hat{{\bf{p}}}\times \left(\nabla \times {\bf{p}}\right)\right]}^{2}/2$, where $\hat{{\bf{p}}}={\bf{p}}/\left|{\bf{p}}\right|$ and *k*_0_ and *k*_2_ penalize the splay and bend distortions (the twist contribution vanishes due to symmetry^31^). The second term represents the surface energy, given by a weak anchoring interaction with ${f}_{{\rm{S}}}=w{\left({\bf{p}}-{{\bf{p}}}_{0}\right)}^{2}/2$, where **p**_0_ is the preferred value for the order parameter at the boundary. We consider cases in which the confining surface is formed by two distinct surfaces, *S*_*α*_ (ExE-boundary) and *S*_*β*_ (VE-boundary), and we take ${{\bf{p}}}_{0}^{\alpha }$ to be tangential to *S*_*α*_ and ${{\bf{p}}}_{0}^{\beta }$ to be normal to *S*_*β*_. If the spatial coordinates are normalized by the characteristic length ${R}_{0}=(3{V}_{0}/4{\uppi})^{1/3}$, equation (1) can be rewritten in the rescaled space *Ω*′ as

2 $$\frac{F[{\bf{p}}]}{{F}_{0}}={\int }_{{\varOmega} {\prime} }{\rm{d}}V{\prime} \left[{|{\bf{p}}|}^{2}+{\left(\frac{\xi }{{R}_{0}}\right)}^{2}{\tilde{f}}_{{\rm{D}}}({\bf{p}},\nabla {\bf{p}})\right]+{\int }_{\partial {{\varOmega} {\prime}} }{\rm{d}}S{\prime} \frac{\lambda }{{R}_{0}}{({\bf{p}}-{{\bf{p}}}_{0})}^{2},$$

where ${F}_{0}=a{R}_{0}^{3}/2$ and ${\widetilde{f}}_{{\rm{D}}}={\left(\nabla {\boldsymbol{\cdot }}{\bf{p}}\right)}^{2}+K{\left[\hat{{\bf{p}}}\times \left(\nabla \times {\bf{p}}\right)\right]}^{2}$ with ${K\equiv {k}_{2}/k}_{0}$. Parameters $\xi \equiv \sqrt{{k}_{0}/a}$ and $\lambda \equiv w/a$ are the correlation and anchoring lengths of the system, respectively, and tune the importance of the distortion and anchoring contributions.

To minimize the free energy in equation (2), we implemented the finite-element method using the FEniCSx library DOLFINx^72^ in Python3. Given the axial symmetry of the system, the mesh (resolution, 0.05) was defined in spherical coordinates in terms of *r* and *ϕ* only, corresponding to a constant-*ϕ* slice of the 3D system. We computed the variation of equation (2) with respect to **p** in the direction of a test function *ψ* to derive its weak formulation. The resulting nonlinear problem was solved using a Newton solver with a relative tolerance of 10^−6^.

### Fitting geometrical parameters to embryo shape

We consider axis-symmetric confining surfaces like the one shown in Fig. 3b, formed by two spherical caps *S*_*μ*_, $\mu =\alpha ,\beta$ representing the ExE–EPI and VE–EPI interfaces, respectively. Each cap is centred at the point (0,*C*_*μ*_) on the symmetry axis and has a radius ${R}_{\mu }\equiv 1/{\kappa }_{\mu }$, where ${\kappa }_{\mu }$ is the curvature. They can be parameterized in spherical coordinates as

3 $$r\left(\theta ;{\kappa }_{\mu },{\gamma }_{\mu }\right)=\frac{\cos \theta }{{\gamma }_{\mu }}+\sqrt{\frac{1}{{\kappa }_{\mu }^{2}}-\frac{{\sin }^{2}\theta }{{\gamma }_{\mu }^{2}}},$$

where ${\gamma }_{\mu }\equiv 1/{C}_{\mu }$, with $\theta \in \left[0,\uppi /2\right]$ for $\mu =\alpha$ and $\theta \in \left[\uppi /2,\uppi \right]$ for $\mu =\beta$ and $\phi \in \left[\mathrm{0,2}\uppi \right)$. To determine the best set of geometrical parameters *R*_*μ*_, *C*_*μ*_ for a given EPI shape, we fit equation (3) to ExE ($\mu =\alpha$) and VE ($\mu =\beta$) boundary data, as described in ref. ^31^. Briefly, we convert the *N*_*μ*_ experimental data points to spherical coordinates, ${\left\{\left({r}_{\mu i}^{\exp },{\theta }_{\mu i}^{\exp },0\right)\right\}}_{i=1\ldots {N}_{\mu }}={\left\{{{\bf{r}}}_{\mu i}^{\exp }\right\}}_{i=1\ldots {N}_{\mu }}={{\bf{r}}}_{\mu }^{\exp }$, and minimize the cost function

4 $$h\left({{\bf{r}}}_{\alpha }^{\exp },{{\bf{r}}}_{\beta }^{\exp };{\kappa }_{\alpha },{\gamma }_{\alpha },{\kappa }_{\beta },{\gamma }_{\beta }\right)=\mathop{\sum }\limits_{\mu =\alpha ,\beta }\mathop{\sum }\limits_{i=1}^{{N}_{\mu }}{\left[\rho \left({{\bf{r}}}_{\mu i}^{\exp };{\kappa }_{\mu },{\gamma }_{\mu }\right)\right]}^{2}$$

and enforcing the constraint $r\left(\uppi /2;{\kappa }_{\alpha },{\gamma }_{\alpha }\right)=r\left(\uppi /2;{\kappa }_{\beta },{\gamma }_{\beta }\right)$. In equation (4), $\rho \left({{\bf{r}}}_{\mu i}^{\exp };{\kappa }_{\mu },{\gamma }_{\mu }\right)={r}_{\mu i}^{\exp }-r\left({\theta }_{\mu i}^{\exp };{\kappa }_{\mu },{\gamma }_{\mu }\right)$ is the residual between the radius of experimental point *i* of boundary *μ* and the fitting function in equation (3) evaluated at that point.

### Image analysis

Preprocessing for machine learning-based membrane segmentation and signal intensity measurement were performed with Fiji^73^. Membrane segmentation and custom model training were performed using Cellpose 2.0 (refs. ^74,75^) GUI or CLI using a bash script. Manual correction of segmentation and cell tracking were performed with napari^76^. Measurements of the angle between the long axes of neighbouring cells, measurements of the cell orientation, analysis of the correlation between signal intensity and cell orientation, and the tissue-scale alignment analysis were performed using custom Python scripts.

### Machine-learning-based membrane segmentation

The segmentation pipeline used to process the 3D images of the membrane signal consists of four steps. In the first step, the source 3D images were preprocessed to generate the membrane channel with an isotropic voxel size. In the second step, custom segmentation models were developed on the Cellpose platform. In the third step, segmentation tasks were performed in batch mode using the Cellpose CLI. In the fourth step, the EPI membrane segmentation was manually selected and corrected in napari. The details of the individual steps are described below.

#### Preprocessing

Airyscan images were binned to 0.1647 × 0.1647 × 0.1600 µm^3^ by averaging 2 × 2 × 1 voxels, followed by isotropic transformations of voxels to a cube with a length of 0.1632 ± 0.0002 µm. To quantify spatial parameters, we set the length of the voxel to 0.1632 µm and ignored the associated error. Images acquired by light-sheet microscope were first cropped to remove background voxels as much as possible and binned to 0.208 ×0.208 ×1.000 µm^3^ by averaging 2 ×2 × 1 voxels, followed by isotropic scaling to 0.351 ± 0.001 µm.

Two channels for E-cadherin and phalloidin signals of the isotropically scaled Airyscan images were combined to generate a ubiquitous membrane channel by summing their signal intensities (Fig. 1a and Extended Data Fig. 1a). Similarly, two channels for the mG and mT signals of the isotropically scaled light-sheet microscopy images were also used to generate a membrane channel (Fig. 1e). These membrane channels were used as inputs for segmentation.

#### Developing custom segmentation models

Since the pretrained models provided by Cellpose 2.0 required an insuperable amount of manual correction for our 3D images, we developed custom segmentation models tailored for Airyscan images and light-sheet microscopy images independently.

To develop models for Airyscan images, preprocessed images and their corresponding ground-truth masks were prepared. In brief, a preprocessed 3D image of an E5.0-stage embryo was resliced into three orthogonal planes (*x*–*y*, *y*–*z* and *x*–*z*), and five slices were extracted in each plane at a 50-slice interval across the entire image. The resulting slices were subjected to 2D segmentation using the Cellpose pretrained model, CPx, followed by manual corrections of all cells in the slice using napari. Following the instructions, two embryo datasets, each consisting of 15 pairs of slices and ground-truth masks, were used to train the neural network, generating ‘AS_model_1’ (Extended Data Fig. 1b). To further improve AS_model_1, we conducted additional training by incorporating two extra embryo datasets into the neural network, resulting in ‘AS_model_2’. Although both models showed comparable performance on images that have high signal-to-noise ratios, AS_model_2 is more robust to the images with various signal-to-noise ratios. Therefore, we used AS_model_2 in this study (Figs. 1, 4 and 5).

Models for light-sheet microscopy images were developed using Cellpose with sparse annotations^77^. The combined membrane stacks at three different time points of a time-lapse images were subjected to 3D segmentation using the CPx model, followed by manual corrections of EPI cells using napari. These ground-truth images were used to train the neural network iteratively, generating ‘LS_model_4’.

#### Batch segmentation

For the 3D segmentation of Airyscan images, segmentation parameters, such as flow_threshold, cellprob_threshold and stitch_threshold, were set to default values, except for the cell diameter, which was set to 60 pixels. AS_model_2 was chosen for this process.

For the 3D segmentation of light-sheet microscopy images, segmentation parameters, such as flow_threshold, cellprob_threshold and stitch_threshold, were set to default values, except for the cell diameter, which was set to 30 pixels. LS_model_4 was selected for this process.

#### Manual correction

Segmentation errors were manually corrected by referring to the original images, and EPI cells were selected and counted in napari using a custom plug-in (napari-segmentation-toolbox). The corrected segmentation masks were saved as ‘.tif’ files for further analysis.

### Neighbouring cell angle measurement

Neighbouring cell angle measurements were performed in Python (v. 3.9) using manually corrected voxel-based segmentation masks. The principal inertia vectors of a label were computed, and the angle between the long axes (primary components) of adjacent labels was measured as the neighbouring cell angle. Neighbouring cells were identified by dilating the label after binarization and computing the overlapping labels by element-wise multiplication. Angle data from the neighbouring cell pairs with overlapping volumes smaller than 20 μm^3^ were excluded from the analysis. The frequency of angle distribution was normalized within each group based on the EPI cell number.

### Mesh-based computations of polarity vectors, surface normals and alignment

To analyse the cell alignment and polarity within the EPI tissue, we first converted the corrected EPI segmentation into a mesh representation using the Python package scikit-image^78^. Each segmented cell was reconstructed as an individual mesh, and a global mesh for the entire EPI region was generated. To ensure consistency in the analysis, all meshes were post-processed to correct face orientations, and only the largest connected component was retained. The final EPI mesh stored cell labels as vertex attributes, enabling cell-specific calculations. The boundary normal vector for a given cell was computed as the average normal vector of all mesh vertices from the EPI mesh associated with that cell. We then used the trimesh library (https://github.com/mikedh/trimesh) to compute various geometric and topological properties, including cell volume, surface area, centroid and principal inertia vectors. Additionally, the centroid of the entire EPI region was determined to facilitate further spatial analyses.

The polarity vector was defined by the largest principal inertia component. Polarity vectors were flipped if the corresponding cell–boundary normal was in the opposite direction or, for interior cells, if the vector was pointing towards the EPI centre. Cell orientation to the tissue boundary was computed for all cells touching the boundary as the angle between the polarity vector and the normal vector, ranging from 0° to 90°, where 0° indicates the perpendicular orientation, whereas 90° indicates the parallel orientation.

### Determination of distal–proximal axis

Imaging data were annotated with at least six points in napari, through which a regression plane approximately separates the boundary between the ExE and VE regions. One point was annotated to denote the distal tip of the EPI. We defined a rotation axis as a line that passes through the EPI centroid and the annotated tip point. Two points were also annotated to indicate the transverse edge of the EPI, the mean position of which was used to establish a rotation plane at angle 0°.

### Weighted nematic average over rotational slices

To construct a 3D nematic vector field representing average cell alignment, we first reoriented the coordinate system such that the *z* axis aligned with the distal–proximal axis, as previously annotated. The spatial coordinates were then rescaled to normalize the EPI volume to unity. At each spatial location ${\bf{r}}=\left(x,y,z\right)$, we extracted the major principal inertia vector $\mathbf{v}\left({\bf{r}}\right)\in {{\rm{R}}}^{3}$ and its corresponding inertia components *λ*_1_ ≤ *λ*_2_ ≤ *λ*_3_ from the occupying cell mesh. The elongation axis was then defined as ${\mathbf{v}}^{{\prime} }\left({\bf{r}}\right)=\eta \left({\bf{r}}\right)\mathbf{v}\left({\bf{r}}\right)$, where the shape anisotropy factor $\eta \left({\bf{r}}\right)=\frac{{\lambda }_{3}-\sqrt{{\lambda }_{1}{\lambda }_{2}}}{{\lambda }_{3}}$ ranged from 0 for spheres up to 1 for infinitely elongated shapes. Next, we defined *M* = 36 equidistant angles *θ*_*m*_, generating rotational slices around the *z* axis. For each slice, we used the rotation matrix *R*(*θ*_*m*_) that maps from the rotational slice to the *x*–*z* plane to obtain elongation axes within the *x*–*z* plane as $\mathbf{v}^{{\prime\prime} }_{m}({\mathbf{r}})=R({\theta }_{m})\mathbf{v}^{\prime} ({R}^{T}({\theta }_{m}){\bf{r}})$ (Fig. 2b). We then computed the weighted Landau–de Gennes *Q* tensor, defined as

$$Q(\mathbf{r})=\frac{1}{M}\sum_{m = 1}^{M} ||\mathbf{v}^{{\prime\prime} }_{m}(\mathbf{r})||\left(\frac{d}{d-1}\frac{\mathbf{v}^{{\prime\prime}}_{m}(\mathbf{r})\otimes \mathbf{v}^{{\prime\prime} }_{m}(\mathbf{r})}{||\mathbf{v}^{{\prime\prime} }_{m}{(\mathbf{r})}||^{2}}-\frac{I}{d-1}\right),$$

for *d* = 3. Finally, we took its principal eigenvector **V**(**r**) as the (unsigned) director field and the corresponding eigenvalue *Λ*(**r**) as the strength of the nematic alignment. This yields the average cell alignment vector **w**(**r**) = *Λ*(**r**)**V**(**r**). For visualization in two dimensions, we evaluated this quantity over a uniform grid in the *x*–*z* plane, displaying only the in-plane components (*w*_*x*_, *w*_*z*_) and additionally using the strength of the nematic alignment $\varLambda ({\mathbf{r}})={||\mathbf{w}}\left({\mathbf{r}}\right){{||}}_{{{\rm{R}}}^{3}}$ as a heat map. Note that the choice between *x*–*z* and *y*–*z* planes is immaterial, as averaging over 36 equidistant rotations ensures rotational invariance and the two planes differ only by a 90° offset. Furthermore, except for the 2D visualization, all analyses were performed in a full 3D space, including directions orthogonal to the chosen 2D planes.

### Generation of rotationally averaged embryo boundary curve

To obtain a rotationally averaged embryo boundary, we first computed the intersection of the EPI mesh with the *M* rotational slice planes defined by angles *θ*_m_. These boundary curves were converted into polar coordinates, with the EPI centroid as the origin. The curves were resampled at equidistant angles, and their radial distances were averaged across all slices to produce a smooth, rotationally averaged boundary. Finally, the averaged boundary was transformed back into Cartesian coordinates for visualization in the *x*–*z* plane.

### Average nematic magnitude

To quantify the overall nematic alignment of one embryo, we defined the average nematic magnitude as ${{{\varLambda }}}_{{\rm{avg}}}=\frac{\int {{\varLambda }}({\rm{r}}){\rm{d}}V}{{{{\varLambda }}}_{\max }\cdot {\rm{v}}{\rm{o}}{\rm{l}}({\varOmega }_{{\rm{avg}}})}$, where *Ω*_avg_ is the 3D volume enclosed by the averaged embryo boundary and *Λ*_max_ denotes the maximal value of the nematic strength factor over all embryos and all positions.

### Cross-embryo averaged nematic averages and boundary curves

For the cross-embryo nematic alignment field, the *Q* tensors were averaged across all embryos as $Q=\frac{1}{K}\sum {Q}_{k}$, where *K* is the number of embryos and *Q*_*k*_ denotes the previously computed *Q* tensors of individual embryos. Similarly, we compute the averaged embryo boundary by averaging with respect to all rotational slices from multiple embryos.

### Fitting the material length scales to the experimental cell elongation field

The nematic alignment tensor captures the cell’s orientation coherence, but not the polarity direction; its magnitude, however, can provide an unsigned estimation of the polarity magnitude, assuming that tail–head inversion is negligibly rare. We define the experimental polarity magnitude as ${p}_{\exp }\equiv {{\varLambda }}/{{{\varLambda }}}_{\max }$, where the strength of the nematic alignment *Λ* is normalized by its maximum across stages *Λ*_max_. This quantity allows us to define the experimental global order as ${P}_{\exp }={\int }_{A}{\rm{d}}z\,{\rm{d}}{x}\,{x}\,{p}_{\exp }/A$, where *A* is the area defined by the average EPI boundaries and the *z* axis.

To determine the model parameters that best characterize a given embryo stage, we fit the average EPI shape as described before, and minimize the free energy functional in equation (2) in such system for the shown range of mechanical parameters *ξ*/*R*_0_ and *λ*/*R*_0_, keeping $K={10}^{-2}$ constant^31^ to obtain the corresponding theoretical field with magnitude *p*. To compare the experimental and theoretical results, we define a set of *N* points **r**_*i*_ belonging to the intersection of the theoretical and experimental embryo shapes. These points must be at a distance greater than 0.05 from the symmetry axis and greater than *d* from the closest boundary, where *d* is given by the mean standard deviation of the experimentally determined average boundary. In this way, we exclude regions in which the average experimental field has low statistics due to the different shapes of the individual embryos. Finally, we calculate the cost function

5 $$C\left(\xi /{R}_{0},\lambda /{R}_{0}\right)=\mathop{\sum }\limits_{i=1}^{N}{\left[p\left({{\bf{r}}}_{i};\xi /{R}_{0},\lambda /{R}_{0}\right)-{p}_{\exp }\left({{\bf{r}}}_{i}\right)\right]}^{2}$$

and identify the set of relative correlation and anchoring lengths, *ξ*/*R*_0_ and *λ*/*R*_0_, where it attains its minimum value.

To establish a comparison between the experimental fields and the corresponding theoretical ones, we define ${{\bf{p}}}_{\exp }\equiv {\bf{w}}/{{\varLambda }}_{\max }$, where **w** is the cell alignment vector (see the ‘Weighted nematic average over rotational slices’ section). Since this quantity represents a nematic field, we consider only its directional information (and not the specific orientation along this direction). Hence, we calculate $|\cos {\widehat{{\bf{p}},{\bf{p}}_{\exp}}}|=|{\bf{p}}\cdot {{\bf{p}}}_{\exp }|/|{\bf{p||}}{{\bf{p}}}_{\exp }|$ as a measure of the degree of alignment of the two fields (Fig. 3g). We also compute the absolute value of the projection of **p**_exp_ and **p** along various paths. We start by rescaling the spatial coordinates as $\widetilde{x}=(x-{x}_{\min })/({x}_{\max }-{x}_{\min })$ and $\widetilde{z}=(z-{z}_{\min })/({z}_{\max }-{z}_{\min })$, where ${x}_{\min }=0$ is the symmetry axis and *x*_max_, *z*_min_ and *z*_max_ correspond to the coordinates of the point at which each path crosses the system boundary. We select three horizontal paths at heights *t*/4, *t*/2 and 3*t*/4 from the distal tip, where *t* is the embryo thickness, and three vertical paths at distances *w*/6, *w*/3 and 2*w*/3 from the symmetry axis, where *w* is the embryo width (Extended Data Fig. 3). Then, we compute the scalar product of the field with the director vector $\hat{x}$ or $\hat{z}$ along the corresponding path, and take the absolute value. In the case of the experimentally determined fields, we do this for each individual embryo and then take the average and standard deviation of the cohort to compare with the theoretical data.

### Statistical analysis

Data were analysed using Python (v. 3.9). Data normality was assessed using the Shapiro–Wilk test. EPI cell number distribution followed a normal distribution, whereas the cellular morphological parameters (cell orientation angles and cell elongation metrics) did not. Comparisons of EPI cell number between various embryo stage groups were performed using one-way analysis of variance followed by Tukey’s post hoc test. For cellular morphological parameters, pairwise comparisons were performed using the Mann–Whitney *U*-test, and comparisons across three or more groups were performed using the Kruskal–Wallis test. For comparisons between two independent groups with normally distributed data, Student’s *t*-test was used. For comparisons against a reference value, one-sample Wilcoxon signed-rank test was used. Linear regression was used to assess relationships between two variables. All statistical tests were two sided. Statistical significance was denoted as NS. *P* > 0.05; *P < 0.05; **P < 0.01; ***P < 0.001.

### Materials availability

All unique/stable reagents generated in this study are available from the corresponding authors with a completed Materials Transfer Agreement.

### Reporting summary

Further information on research design is available in the Nature Portfolio Reporting Summary linked to this article.

## Online content

Any methods, additional references, Nature Portfolio reporting summaries, source data, extended data, supplementary information, acknowledgements, peer review information; details of author contributions and competing interests; and statements of data and code availability are available at 10.1038/s41567-026-03176-9.

## Supplementary information

Reporting Summary

Supplementary Table 1 Genotyping primers and PCR product sizes.

Supplementary Video 1 Light-sheet live microscopy reveals EPI cell orientation dynamics (Fig. 1). Time-lapse images of a Sox2-Cre;mT embryo developing in 3D-geec. The equatorial plane of the embryo is shown in Fig. 1e. Green, memb-GFP in EPI cells; magenta, memb-Tomato in other cell types. Time, hours:minutes. Scale bars, 20 μm.

## Source data

Source Data Figs. 1–6 Statistical source data for Figs. 1b–d,f, 2d, 3f,h,i,l, 4b,c,e,f, 5b,d,f,h and 6b,e.

Source Data Extended Data Figs. 1, 5 and 6 Statistical source data for Extended Data Figs. 1c,d, 5a,c,e and 6a,b,e,f.

## Data Availability

Raw image data are available from the corresponding authors upon request. Source data are provided with this paper.

## Code availability

The code is available at GitHub (https://git.embl.org/guruciag/boundary-guided-epiblast-patterning, https://github.com/SteffenPL/EpiblastOrientationImageAnalysis, https://github.com/SteffenPL/EpiblastOrientationMaps and https://github.com/AnniekStok/napari-segmentation-toolbox).
