# Supplementary material for: Boundary-guided cell alignment drives mouse epiblast maturation
Source: Nat Phys. 2026 Apr 1;22(3):461–73. doi: 10.1038/s41567-026-03176-9 (PMC13046470; doi:10.1038/s41567-026-03176-9)
Supplement: Supplementary file 1 — Reporting Summary [file 41567_2026_3176_MOESM1_ESM.pdf]

nature portfolio

Corresponding author(s): Takafumi Ichikawa, Pamela C. Guruciaga, Anna Erzberger

Last updated by author(s): Jan 1, 2026



# Reporting Summary

Nature Portfolio wishes to improve the reproducibility of the work that we publish. This form provides structure for consistency and transparency in reporting. For further information on Nature Portfolio policies, see our Editorial Policies and the Editorial Policy Checklist.

## Statistics

For all statistical analyses, confirm that the following items are present in the figure legend, table legend, main text, or Methods section.

| n/a | Confirmed | |
|---|---|---|
| ☐ | ☒ | The exact sample size ($n$) for each experimental group/condition, given as a discrete number and unit of measurement |
| ☐ | ☒ | A statement on whether measurements were taken from distinct samples or whether the same sample was measured repeatedly |
| ☐ | ☒ | The statistical test(s) used AND whether they are one- or two-sided<br>*Only common tests should be described solely by name; describe more complex techniques in the Methods section.* |
| ☒ | ☐ | A description of all covariates tested |
| ☐ | ☒ | A description of any assumptions or corrections, such as tests of normality and adjustment for multiple comparisons |
| ☐ | ☒ | A full description of the statistical parameters including central tendency (e.g. means) or other basic estimates (e.g. regression coefficient) AND variation (e.g. standard deviation) or associated estimates of uncertainty (e.g. confidence intervals) |
| ☐ | ☒ | For null hypothesis testing, the test statistic (e.g. $F$, $t$, $r$) with confidence intervals, effect sizes, degrees of freedom and $P$ value noted<br>*Give P values as exact values whenever suitable.* |
| ☒ | ☐ | For Bayesian analysis, information on the choice of priors and Markov chain Monte Carlo settings |
| ☒ | ☐ | For hierarchical and complex designs, identification of the appropriate level for tests and full reporting of outcomes |
| ☐ | ☒ | Estimates of effect sizes (e.g. Cohen's $d$, Pearson's $r$), indicating how they were calculated |

*Our web collection on statistics for biologists contains articles on many of the points above.*

## Software and code

Policy information about availability of computer code

| | |
|---|---|
| Data collection | napari (0.4.16, https://github.com/napari/napari)<br>napari-label-interpolator (https://github.com/brisvag/napari-label-interpolator)<br>napari-simpleitk-image-processing (0.4.6, https://github.com/haesleinhuepf/napari-simpleitk-image-processing)<br>napari-segmentation-correction (https://github.com/AnniekStok/napari-segmentation-correction)<br>Cellpose 2.0 (https://github.com/MouseLand/cellpose)<br>Fiji (ImageJ 1.54g, https://imagej.net/software/fiji/) |
| Data analysis | Custom code (https://git.embl.de/guruciag/boundary-guided-epiblast-patterning) was used to solve the system of partial differential equations and analyse numerical and experimental results. Custom code (https://github.com/SteffenPL/EpiblastOrientationImageAnalysis, https://github.com/SteffenPL/EpiblastOrientationMaps) was used to analyse 3D segmentation data and generate tissue-scale cell alignment maps. |

For manuscripts utilizing custom algorithms or software that are central to the research but not yet described in published literature, software must be made available to editors and reviewers. We strongly encourage code deposition in a community repository (e.g. GitHub). See the Nature Portfolio guidelines for submitting code & software for further information.

## Data

Policy information about availability of data

All manuscripts must include a data availability statement. This statement should provide the following information, where applicable:

- Accession codes, unique identifiers, or web links for publicly available datasets
- A description of any restrictions on data availability
- For clinical datasets or third party data, please ensure that the statement adheres to our policy

Source data for figures are provided with this paper. Raw image data are available from the corresponding authors upon request.

## Research involving human participants, their data, or biological material

Policy information about studies with human participants or human data. See also policy information about sex, gender (identity/presentation), and sexual orientation and race, ethnicity and racism.

| | |
|---|---|
| Reporting on sex and gender | n/a |
| Reporting on race, ethnicity, or other socially relevant groupings | n/a |
| Population characteristics | n/a |
| Recruitment | n/a |
| Ethics oversight | n/a |

Note that full information on the approval of the study protocol must also be provided in the manuscript.

# Field-specific reporting

Please select the one below that is the best fit for your research. If you are not sure, read the appropriate sections before making your selection.

☒ Life sciences ☐ Behavioural & social sciences ☐ Ecological, evolutionary & environmental sciences

For a reference copy of the document with all sections, see nature.com/documents/nr-reporting-summary-flat.pdf

# Life sciences study design

All studies must disclose on these points even when the disclosure is negative.

| | |
|---|---|
| Sample size | Sample size for each experiment was determined by the number of embryos available from one pregnant female, as experimental constraints. No statistical method was used to predetermine sample size. The experiment was independently repeated at least three times. |
| Data exclusions | In Figure 1 and 5, embryo image data with segmentation errors at an unmodifiable level were excluded from the analysis. In Figure 6, after 16 hours of culture, embryos were excluded from analysis if they met either of the following criteria: (1) failure to show proximal-distal axis elongation, or (2) epiblast cell number below 50. These criteria were pre-established to ensure analysis of developing embryos. |
| Replication | The experimental findings were reproduced in three independent experiments. Each experiment was performed on a different day using embryos obtained from different female mice. |
| Randomization | To ensure unbiased comparison, embryos were assigned to control and experimental groups using a balanced distribution method based on their morphological ranking at the embryo recovery. For example, if embryos were ranked from 1 to 8 based on their developmental progression, they would be distributed as (1,4,5,8) and (2,3,6,7), ensuring that each group contained embryos from across the full range of variability. Furthermore, the assignment of these balanced groups to either control or experimental conditions was determined randomly, adding an additional layer of randomization to minimize any potential bias. |
| Blinding | In Figure 5, blinding was implemented by annotating embryo genotype information after the image data analysis was done. In Figure 6, blinding was not possible due to the nature of the experimental manipulations, which were visually distinct. However, to minimize bias, quantitative analyses were performed using automated image processing whenever possible. |

# Reporting for specific materials, systems and methods

We require information from authors about some types of materials, experimental systems and methods used in many studies. Here, indicate whether each material, system or method listed is relevant to your study. If you are not sure if a list item applies to your research, read the appropriate section before selecting a response.

## Materials & experimental systems

| n/a | Involved in the study | |
|---|---|---|
| ☐ | ☒ | Antibodies |
| ☒ | ☐ | Eukaryotic cell lines |
| ☒ | ☐ | Palaeontology and archaeology |
| ☐ | ☒ | Animals and other organisms |
| ☒ | ☐ | Clinical data |
| ☒ | ☐ | Dual use research of concern |
| ☒ | ☐ | Plants |

## Methods

| n/a | Involved in the study | |
|---|---|---|
| ☒ | ☐ | ChIP-seq |
| ☒ | ☐ | Flow cytometry |
| ☒ | ☐ | MRI-based neuroimaging |

# Antibodies

| | |
|---|---|
| Antibodies used | Primary antibodies:<br>- Mouse mAb Anti-Oct3/4 (Santa Cruz Biotechnology, sc-5279 AF647) used at 1:100.<br>- Mouse mAb Anti-E-cadherin (BD Biosciences, 560064) used at 1:100.<br>- Rat mAb Anti-Mouse CD29 (9EG7) (BD Biosciences, 553715), used at 1:100.<br>- Rabbit mAb Anti-Phosphorylated p44/42 MAPK (ERK1/2) (Cell Signaling 4370) used at 1:100.<br>- Rabbit pAb Anti-Laminin (Novus Biologicals, NB300-144) used at 1:200.<br>- Rabbit mAb Anti-Phosphorylated Ezrin, Radixin, Moesin (ERM) (Cell Signaling, 3726) used at 1:200.<br>- Rat mAb Anti-Podocalyxin (R&D Systems, MAB1556) used at 1:200.<br>- Laminin chain specific antibodies were used as described previously (Kim et al. 2022 Development)<br><br>Secondary antibody:<br>- Donkey anti-rabbit IgG Alexa Fluor Plus 488 (Invitrogen, A32790) used at 1:200.<br>- Donkey anti-rabbit IgG Alexa Fluor Plus 555 (Invitrogen, A32794) used at 1:200.<br>- Donkey anti-rat IgG Alexa Fluor Plus 488 (Invitrogen, A48269) used at 1:200. |
| Validation | Antibody validations were performed by antibody suppliers or were published in earlier studies. Relevant articles is:<br>Ichikawa, T., Zhang, H. T., Panavaite, L., Erzberger, A., Fabrèges, D., Snajder, R., Wolny, A., Korotkevich, E., Tsuchida-Straeten, N., Hufnagel, L., Kreshuk, A., & Hiiragi, T. (2022). An ex vivo system to study cellular dynamics underlying mouse peri-implantation development. Developmental cell, 57(3), 373–386.e9. https://doi.org/10.1016/j.devcel.2021.12.023 |

# Animals and other research organisms

Policy information about studies involving animals; ARRIVE guidelines recommended for reporting animal research, and Sex and Gender in Research

| | |
|---|---|
| Laboratory animals | This study used laboratory mice (Mus musculus), F1 hybrid strain between C57BL/6NCrSlc and C3H/HeSlc (B6C3F1/Slc), purchased from Japan SLC. Transgenic animals used in this study were described in the manuscript. All mice were maintained in specific pathogen-free conditions with a 14-10 hours light-dark cycle and used for experiments at the age of 8 to 30 weeks. |
| Wild animals | n/a |
| Reporting on sex | This study used peri-implantation and early post-implantation embryos before apparent sexual differentiation occurs. |
| Field-collected samples | n/a |
| Ethics oversight | All animal work was performed in the Institute of Laboratory Animals (ILA), with permission from the Animal Research Committee, Graduate School of Medicine (approved number, MedKyo 23065) and the Committee for Safety Control of Recombinant DNA Experiments, Kyoto University (approved number, 230029). ILA is operated according to the Regulations on Animal Experimentation at Kyoto University. |

Note that full information on the approval of the study protocol must also be provided in the manuscript.

## Plants

| | |
|---|---|
| Seed stocks | n/a |
| Novel plant genotypes | n/a |
| Authentication | n/a |
